# Phenotypic Traits, SSR Core Primer Screening, and Genetic Diversity Analysis of *Toxicodendron vernicifluum* From Different Seed Sources in Yunnan, China

**DOI:** 10.1002/ece3.71794

**Published:** 2025-07-14

**Authors:** Huiping Zeng, Xingze Li, Jiayu Feng, Cai Wang, Dan Zong, Tao Jiang, Xinglan Wei, Qiong Dong

**Affiliations:** ^1^ College of Forestry Southwest Forestry University Kunming Yunnan China; ^2^ Key Laboratory for Forest Genetic and Tree Improvement & Propagation in Universities of Yunnan Province Southwest Forestry University Kunming China; ^3^ Key Laboratory of State Forestry Administration on Biodiversity Conservation in Southwest China Southwest Forestry University Kunming China; ^4^ School of Biology and Food Engineering Southwest Forestry University Kunming Yunnan China; ^5^ Science and Technology Department Southwest Forestry University Kunming Yunnan China

**Keywords:** genetic diversity, phenotypic traits, SSR primers, *Toxicodendron vernicifluum*, Yunnan Province

## Abstract

Yunnan Province in China, with its unique natural conditions, has nurtured abundant yet fragile biodiversity resources. The *Toxicodendron vernicifluum* is one of the important tree species in Yunnan, valued for its ecological, economic, and medicinal significance. However, due to a lack of related research and limited germplasm resources, breeding and propagation of the *T*. *vernicifluum* have lagged behind other tree species. To address this shortcoming, we collected 36 samples of *T*. *vernicifluum* germplasm from six representative populations in different regions of Yunnan Province. Combining phenotypic traits, we used 24 selected primer pairs to analyze the genetic diversity and genetic structure of all *T. vernicifluum* samples. The results showed that seeds from the NLS and ZYG populations performed best. Among all traits, seed length exhibited the greatest variation and highest plasticity. The average values for the Shannon information index (*I*), expected heterozygosity (He), and polymorphism information content (PIC) of the 24 loci were 0.377, 0.230, and 0.257, respectively, while the average inbreeding coefficient within populations (Fis) was −0.103 (Fis > 0). The ZYG population showed the highest genetic diversity, indicating that the *T. vernicifluum* has accumulated a large amount of genetic variation during its long‐term evolution. AMOVA analysis revealed that 47% of the genetic variation originated within populations. The 36 *T. vernicifluum* families were divided into three groups, and the six populations were subdivided into three subgroups. This study, based on phenotypic traits and SSR molecular markers, analyzes the genetic diversity of *T. vernicifluum* resources from different provenances in Yunnan Province, providing a theoretical reference for discovering elite genetic resources and selecting hybrid parents for *T. vernicifluum* breeding.

## Graphical abstract

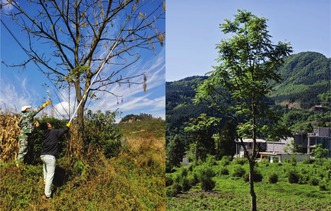

## 1 Introduction

In the more ecologically fragile areas, such as Yunnan, China, forest degradation or forest damage can often be seen (Yin et al. 2024). It is usually manifested as a decrease in stand density (Lee et al. 2023), slow growth of forest trees (Liu et al. 2024), and a reduction in the canopy portion of forest trees (Zhang et al. 2023), etc. These have caused certain impacts on both planted forests and natural forests, including causing the weakening or disappearance of their ecological, economic, and social values, among others. Therefore, the conservation and utilization of forest resources have received more and more attention. As genetic diversity is the key to the health and stability of populations, studying and evaluating the genetic traits of forest tree germplasm in various aspects will largely promote our understanding of forest tree resources, and on this basis, we will be able to better protect and utilize them in the future (Zhai et al. 2024).

*Toxicodendron vernicifluum* (Stokes) F. A. Barkle, as one of China's very important forest resources, is a deciduous tree species of the genus *Toxicodendron* under the family Anacardiaceae. We call it the lacquer tree. It is usually diploid and can reach up to 20 m in height. It is mainly distributed in the Qinba Mountains and the Yunnan‐Guizhou Plateau, with concentrated distribution in Shanxi, Shaanxi, Hubei, Sichuan, Chongqing, Yunnan, and other regions (Shang et al. 2024). The *T. vernicifluum* possesses a very rich reservoir of excellent genetic resources (Li et al. 2017). It has great medicinal, economic, and ecological value (Veenhoven et al. 2023; Xiong et al. 2022). Of these, several recent studies deserve special emphasis, demonstrating its great medical value: Ultrasound‐assisted antler *T. vernicifluum* ‐mediated green synthesis of gold nanoparticles can be used for the treatment of lung cancer (Zhang et al. 2024); *T. vernicifluum* extracts exhibit potent antioxidant and antibacterial effects against drug‐resistant microorganisms and are expected to be compounds for the treatment of drug‐resistant infections in the future (Nafiseh et al. 2024); *T. vernicifluum* acids can improve neurological deficits in traumatic brain injury through anti‐iron death and anti‐inflammation (Liu et al. 2023). Therefore, it is of great significance to analyze the genetic evolution of *T. vernicifluum* .

However, in recent years, due to the influence of market and other external conditions, precious *T. vernicifluum* resources have suffered damage. The long history of cultivation has also led to the degradation of existing varieties, reduced yields, and lowered quality. Therefore, there is an urgent need to identify and utilize a group of high‐quality germplasm resources to improve and enhance the current cultivated varieties. At present, research on *T. vernicifluum* mainly focuses on the distribution of germplasm resources (Wang, Zhou, et al. 2024), extraction and synthesis of internal components (Harigaya et al. 2007), and germplasm evaluation and genetic analysis of *T. vernicifluum* resources in the Qinba area (Wang et al. 2022). However, studies on the genetic diversity and spatial distribution of Yunnan *T. vernicifluum* populations are rare. Genetic diversity analysis is one of the main components of germplasm resource research and is also a prerequisite for the innovative utilization of germplasm resources and the breeding of new varieties (Luo et al. 2023). Studies on species genetic diversity can be evaluated through two approaches: phenotypic traits and molecular markers (Wang, Li, et al. 2024).

Phenotypic trait assessment includes the use of tree characteristics and seed characteristics, which can directly or indirectly reflect trends or correlations of genetic variation in forest trees (Climent et al. 2024). Due to their ease of evaluation, these traits are often used as evaluation indicators for forest economic traits, genetic traits, and so on (Benavides et al. 2022). Seed trait characteristics often vary greatly among different provenances, and their different traits are crucial for their dispersal, deposition, and germination. Especially in extreme ecological environments, the structure of plant communities is largely influenced by environmental selective effects on seeds; with varying environments, seed phenotypic variation also differs (Subodh et al. 2024). If the genetic correlation among phenotypic traits of trees is considered, it should be noted that the genetic correlation among traits may vary due to different factors such as human activities and environmental selection. In addition, the interaction between genotype and environment may also affect the manifestation of tradeoffs. Since the plasticity of different phenotypic traits may vary, the correlation among traits may weaken or even change from positive to negative under certain conditions (Benavides et al. 2021; Sole‐Medina et al. 2022; Schneider 2022).

SSR (simple sequence repeat) marker assessment evaluates genetic diversity at the molecular level. SSR, also known as microsatellites, is widely used in studies of biological genetic diversity, resource phylogenetic relationships, variety identification, and genetic map construction (Chalbi et al. 2023; Zhao et al. 2023; Chikh‐Rouhou et al. 2021). Compared with several other markers such as RAPD, ISSR, and AFLP, SSR markers are favored for their codominant nature. Furthermore, this marker technology possesses advantages such as high information content, strong reproducibility, ease of operation, and low cost, allowing it to stand out in practical applications across multiple fields (Chikh‐Rouhou et al. 2021; Ferrão et al. 2015; Varshney et al. 2005). In addition, constructing SSR molecular fingerprint databases based on fluorescence capillary electrophoresis platforms offers high sensitivity, accurate data reading, and high throughput (Franco et al. 2024). This can assist in genetic map construction, target trait gene mapping, genetic diversity analysis, and gene positional cloning, and it is also a widely used molecular marker technology in studies such as conservation genetics and phylogeography (Ma et al. 2020).

This study aims to clarify the genetic structure and genetic diversity of *T. vernicifluum* resources in Yunnan Province, China, and to provide standardized guidance for the rational utilization of *T. vernicifluum* resources. Through comprehensive analyses based on phenotypic traits and SSR molecular markers, the genetic structure and genetic diversity of 36 *T. vernicifluum* families from six populations across three provenances in Yunnan Province were investigated. The goal is to provide a scientific basis and reference guidelines for the identification, research, and application of *T. vernicifluum* varieties. By offering technical support, the study promotes the collection, identification, classification, preservation, and selection of superior parents of *T. vernicifluum* germplasm resources. Additionally, this research aims to protect existing *T. vernicifluum* resources, reasonably develop natural *T. vernicifluum* forests, and explore their potential economic value. In summary, the study mainly seeks to address the following questions: (1) How different are the phenotypic traits of *T. vernicifluum* from different seed sources in Yunnan Province? What are the correlations among traits? Which *T. vernicifluum* family lines are of higher quality? (2) Based on the developed SSR primers of Anacardiaceae, what are the primers suitable for *T. vernicifluum* in Yunnan Province? (3) Based on SSR molecular marker technology, understand the genetic diversity and population structure of 36 representative *T. vernicifluum* germplasm from different seed sources in Yunnan Province, and compare the genetic characteristics of different subpopulations.

## 2 Materials and Methods

### 2.1 Sampling and Study Area

**FIGURE 1:**
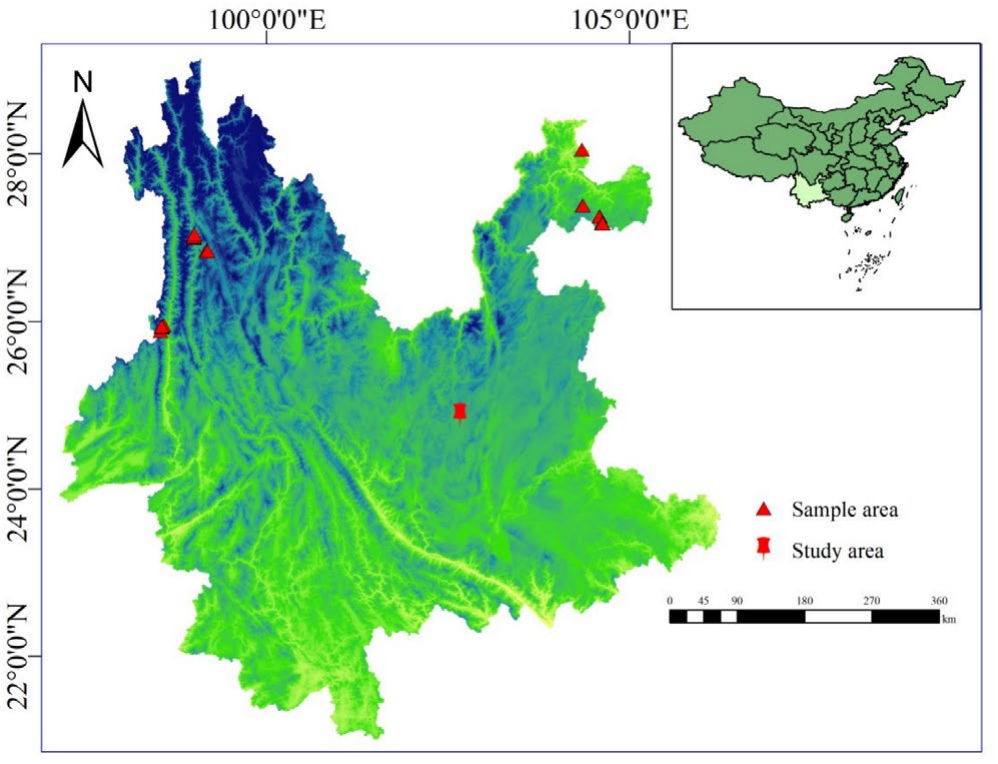
Map of sampling areas and study areas for 36 representative *Toxicodendron vernicifluum* materials in Yunnan Province, China.

As shown in Figure 1, 36 representative samples of *Toxicodendron vernicifluum* were collected in Zhaotong City, Yunnan Province; Nujiang Lisu Autonomous Prefecture, Yunnan Province; and Diqing Tibetan Autonomous Prefecture, Yunnan Province, which are rich in *T. vernicifluum* resources, through data collection and field surveys. Sample areas were selected in Niuchang Town, Haizi Town, and Yigu Town of Zhaotong City; Luzhang Town and Hexi Town of Nujiang Prefecture; and Weideng Town of Diqing Prefecture, with a total of three seed sources and six populations. The study area was located at the Southwest Forestry University of Kunming City, Yunnan Province (E102° 46′, N25° 03′), which is situated on the Yunnan–Guizhou Plateau in the subtropical highland monsoon climatic zone, with an elevation of 1964 m. It is characterized by a short frosty period and a mild climate.

### 2.2 *Toxicodendron vernicifluum* Materials and SSR Primer Materials

#### 2.2.1 *Toxicodendron vernicifluum*

**FIGURE 2:**
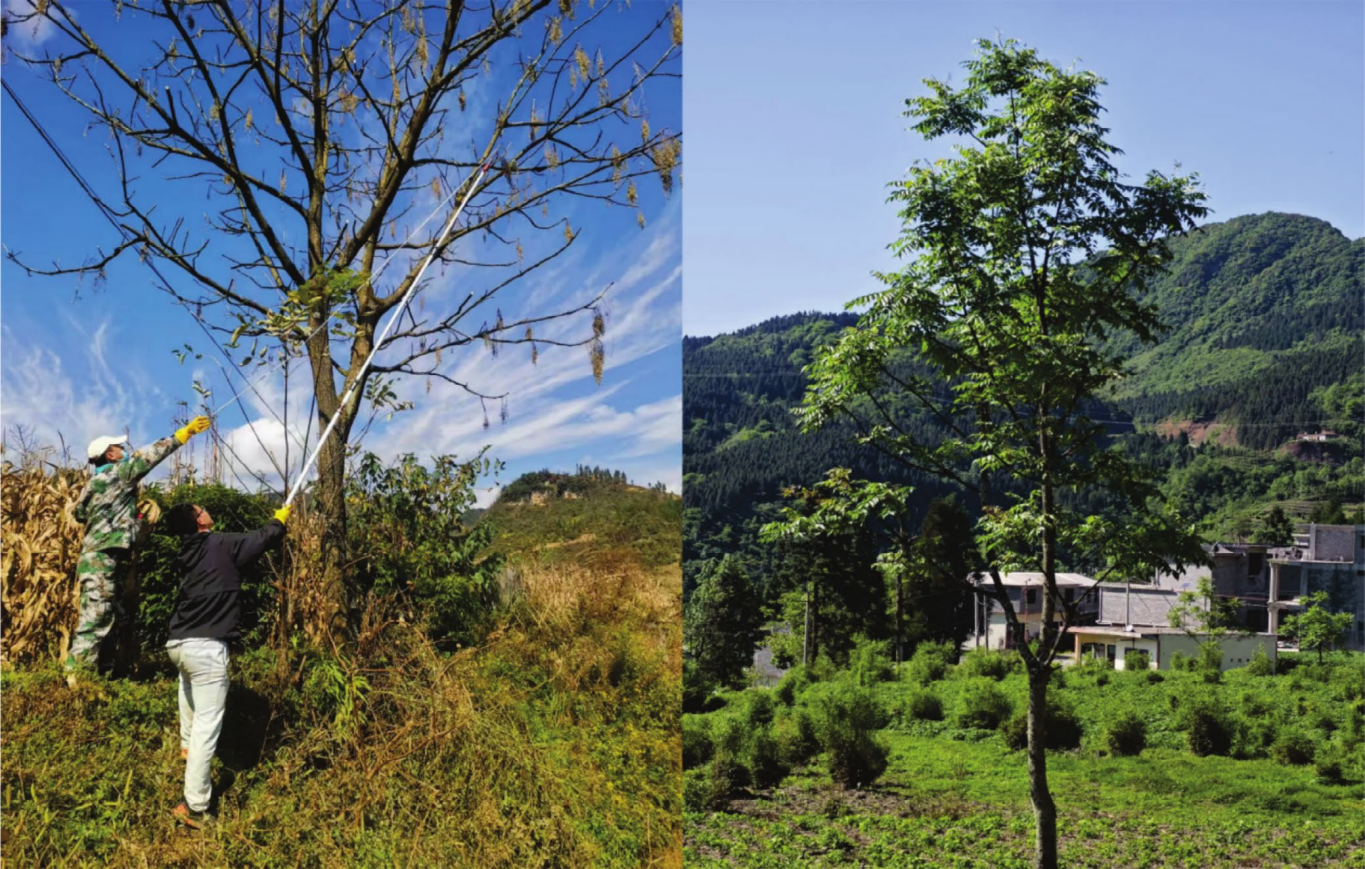
Mature morphology in autumn and leaf‐spreading morphology of *Toxicodendron vernicifluum* in spring.

The mature morphology of *T. vernicifluum* in autumn as well as the leaf‐spreading morphology of *T. vernicifluum* in spring are shown in Figure 2. The material of *T. vernicifluum* used in this study was identified by Qi Lin, and the specimen was deposited in the Herbarium of the Institute of Botany, Chinese Academy of Sciences, under the number 02245732 (https://www.cvh.ac.cn/spms/detail.php?id=e6fd160c). During the mature fruit period of *T. vernicifluum* from September 2023 to November 2023, a sufficient quantity of seeds from 36 families of *T. vernicifluum* was collected in Niuchang Town, Haizi Town, and Yigu Town of Zhaotong City; Luzhang Town and Hexi Township of Nujiang Prefecture; and Weideng Township of Diqing Prefecture, with proper labeling for each family. In the spring of the following year (April 2024 to June 2024), during the growth period and leaf spreading of the species, we again went to the location of the six populations from which seeds had been taken, collected 36 young leaves of *T. vernicifluum* families, and dehydrated them with silica gel to bring them back to the test room and preserve them in the refrigerator at −80°C for spare use. The information about the sources of the 36 samples is shown in Table 1, of which the ZNC and ZHZ populations are between 1700 and 1800 m, the ZYG and NLS are basically between 1800 and 2000 m, and the NHX and DWD populations are above 2000 m. The slopes are all medium or downhill, and most of the slopes have an easterly aspect.

**TABLE 1:** Source of material for 36 *Toxicodendron vernicifluum* samples.

Sample	Sampling location	Longitude and latitude	Altitude/m	Slope position	Slope orientation
NC‐1	Niuchang Town, Zhenxiong County, Zhaotong City, Yunnan Province, China (ZNC)	27°47′24.3″ N, 104°96′43.1″ E	1748	Downhill	East
NC‐2		28°24′23.4″ N, 104°35′33.4″ E	1730	Middle slope	East
NC‐3		27°47′54.3″ N, 104°59′32.1″ E	1724	Downhill	East
NC‐4		27°47′65.4″ N, 104°59′51.3″ E	1730	Downhill	East
NC‐5		27°47′31.7″ N, 104°59′51.4″ E	1730	Downhill	East
NC‐6		27°47′41.2″ N, 104°95′21.1″ E	1730	Downhill	East
HZ‐1	Haizi Town, Yiliang County, Zhaotong City, Yunnan Province, China (ZHZ)	27°60′71.2″ N, 104°36′75.5″ E	1710	Middle slope	West
HZ‐2		27°60′72.6″ N, 104°36′82.9″ E	1720	Middle slope	West
HZ‐3		27°60′75.7″ N, 104°36′80.6″ E	1729	Middle slope	West
HZ‐4		27°60′74.9″ N, 104°36′84.1″ E	1742	Middle slope	Southwest
HZ‐5		27°60′67.4″ N, 104°36′86.4″ E	1735	Middle slope	Southwest
YG‐1	Yigu Town, Zhenxiong County, Zhaotong City, Yunnan Province, China (ZYG)	27°39′64.5″ N, 104°63′65.4″ E	1829	Middle slope	Northeast
YG‐2		27°39′34.5″ N, 104°63′43.5″ E	1850	Middle slope	East
YG‐3		27°39′54.3″ N, 104°62′95.4″ E	1860	Middle slope	East
YG‐4		27°39′45.6″ N, 104°62′94.5″ E	1850	Middle slope	East
YG‐5		27°39′54.6″ N, 104°63′65.4″ E	1830	Middle slope	East
YG‐6		27°39′13.5″ N, 104°63′64.3″ E	1830	Middle slope	East
YG‐7		27°39′13.7″ N, 104°63′71.2″ E	1830	Middle slope	East
YG‐8		27°39′13.7″ N, 104°63′76.5″ E	1810	Middle slope	East
LS‐1	Luzhang Town, Lushui City, Nujiang Prefecture, Yunnan Province, China (NLS)	25°96′34.5″ N, 98°75′73.5″ E	2065	Middle slope	South
LS‐2		26°03′54.4″ N, 98°75′37.5″ E	1764	Downhill	Northeast
LS‐3		26°01′42.4″ N, 98°76′35.4″ E	2020	Downhill	East
LS‐4		26°01′34.4″ N, 98°76′43.4″ E	2036	Downhill	North
LS‐5		26°01′43.2″ N, 98°76′32.1″ E	1937	Middle slope	Northeast
LS‐6		26°01′43.5″ N, 98°76′89.7″ E	1938	Middle slope	East
LS‐7		26°01′65.7″ N, 98°76′23.7″ E	1940	Downhill	North
HX‐1	Hexi Township, Lamping County, Nujiang Prefecture, Yunnan Province, China (NHX)	26°95′63.5″ N, 99°31′56.7″ E	2454	Middle slope	East
HX‐2		26°95′28.7″ N, 99°31′34.3″ E	2415	Middle slope	Northwest
HX‐3		26°95′76.2″ N, 99°31′34.3″ E	2415	Middle slope	Northwest
HX‐4		26°94′59.8″ N, 99°32′78.7″ E	2670	Middle slope	Southeast
HX‐5		26°94′98.2″ N, 99°32′61.2″ E	2693	Middle slope	Northeast
HX‐6		26°94′39.8″ N, 99°32′69.8″ E	2706	Middle slope	Northeast
WD‐1	Wideng Township, Wisi County, Diqing Prefecture, Yunnan Province, China (DWD)	27°09′72.5″ N, 99°13′83.2″ E	2400	Middle slope	Northeast
WD‐2		27°09′93.2″ N, 99°13′34.3″ E	2433	Middle slope	North
WD‐3		27°13′42.5″ N, 99°13′55.4″ E	2346	Middle slope	Northeast
WD‐4		27°14′23.4″ N, 99°13′56.6″ E	2355	Middle slope	Northeast

#### 2.2.2 SSR Primers

All 160 pairs of SSR primers screened for *T. vernicifluum* were based on the existing research base and were cited from the relevant literature on plant species identical or similar to Anacardiaceae (Yuichiro and Atsushi 2010; Hsu et al. 2013; Li et al. 2018; Vu et al. 2018; Guo et al. 2019; Ruan et al. 2020; Cai et al. 2024). Information on the 160 pairs of primers is given in Appendix S1. SSR primers were synthesized by Shanghai Shenggong Biotechnology Co. Ltd.

### 2.3 Phenotypic Traits, Genomic DNA Extraction, and SSR Primer Screening Methods in *Toxicodendron vernicifluum*

#### 2.3.1 Phenotypic Traits Evaluated

At the time of seed collection of 36 *T. vernicifluum* family lines from September 2023 to November 2023, tree height (m) and clear length (m) were measured with a Brulé height gauge, diameter of chest (cm) was measured by a breast diameter ruler, and tree age (a) was estimated by using the interview estimation method. The collected *T. vernicifluum* seeds were sun‐dried in December 2023, and all the seeds collected from each family line were mixed. Thirty seeds were randomly selected and replicated 30 times, and the seed length, seed breadth, and seed thickness were measured using vernier calipers with an accuracy of 0.01 mm. The seed length‐to‐width ratio, coefficient of variation (%), and plasticity index were also calculated (Tian et al. 2021; Zhu et al. 2022):

(1) Seed length‐to‐width ratio=seed length/seed breadth

(2) $$\begin{array}{c} \text{Coefficient of variation}\;\left(\%\right)\\ \;=\left(\text{Standard deviation of each trait}/\text{mean of each trait}\right)\\ \;\times 100\% \end{array}$$

(3) $$\text{Plasticity index}=\left(\text{maximum}-\text{minimum}\right)/\text{maximum}$$

#### 2.3.2 DNA Extraction and PCR Amplification

*T. vernicifluum* genomic DNA was extracted by using the Polysaccharide and Polyphenol Plant Genomic DNA Extraction Kit (Centrifugal Column Type) Tiangen Biochemical Technology (Beijing) Co. refer to kit instructions for specific steps. The extracted DNA was tested for concentration and quality by agarose gel (1%) electrophoresis and NanoDrop 8000 Ultra‐Micro Spectrophotometer (Thermo Fisher Scientific, USA), respectively, and then stored in a refrigerator at −20°C.

The SSR‐PCR reaction system with a total volume of 25 μL contained 12.5 μL of 2 × PCR‐Mix, 9.5 μL of ddH_2_O, 1 μL each of SSR forward and reverse primers, and 1 uL of DNA template. The PCR amplification procedure was as follows: predenaturation at 94°C for 4 min, denaturation at 94°C for 30 s, Tm (gradient) redenaturation for 30 s, extension at 72°C for 1 min for 35 cycles, extension at 72°C for another 10 min, and storage at 4°C. After the PCR reaction, the amplification products were detected by fluorescence capillary electrophoresis. Based on the above reaction system and reaction procedure, two samples were randomly selected from each *T. vernicifluum* population, totaling 12 samples. From the selected 160 primers to be screened, after initial and re‐screening (Appendix S2), the primers with high stability and polymorphism were screened, and 24 pairs of primers were screened out for the amplification of all the *T. vernicifluum* samples after the detection of the amplification results (Table 4).

### 2.4 Data Analysis

#### 2.4.1 Data Processing of Phenotypic Indicators of *Toxicodendron vernicifluum*

Georeferenced maps of sampling and test sites were obtained using ArcMap 10.6 (ESRI., Redlands, CA, USA). Statistical analyses were performed using SPSS 27.0 (SPSS Inc., Chicago, IL, USA). Correlation analysis, principal component analysis, and graphing were performed using Origin 2021 (OriginLabCo., Northampton, MA, USA).

#### 2.4.2 SSR Molecular Marker Data Processing

The amplification of 36 *T. vernicifluum* DNA samples was completed with 24 pairs of primers selected and detected by capillary electrophoresis, and the capillary electrophoresis peak graph was obtained. The sequence size of the amplification product of each DNA sample was read from the peak plot by GeneMarker analysis software, and the genotype data were counted and organized using Excel 2016, and the data format was converted according to the requirements of the analysis software. The polymorphic information index (PIC) was calculated for each site by Cervus 3.0.7 software. GenAlEx 6.41 software was used to calculate the number of alleles (Na), effective number of alleles (Ne), Shannon's information index (*I*), observed heterozygosity (Ho), expected heterozygosity (He), fixation index (*F*), intrapopulation inbreeding coefficient (Fis), total intrapopulation inbreeding coefficient (Fit), population divergence coefficient (*F*_st_), gene flow (*Nm*), Nei's genetic distance between samples or populations (*D*), molecular analysis of variance (AMOVA), and principal coordinate analysis (PCoA) were also performed (Peakall and Smouse 2006), while individual and population UPGMA tree clustering maps of *Toxicodendron vernicifluum* based on genetic distances were drawn using Powermarker and MEGA 6.0 software (Koichiro et al. 2013). Genetic similarity coefficients and genetic distances between populations and individuals were calculated using POPGENE 32 software. Genetic structure analysis was performed on 36 *T. vernicifluum* resources using STRUCTURE 2.3.4 software (Evanno et al. 2005), setting K = 2–10, Burnin period 10,000, MCMC (Markov Chain Monte Carlo) as 100,000, and each K value was run 15 times, and then the best K value was determined on the Structure Harvester website. The best K value was determined, and finally, subpopulations were divided according to the run with the largest likelihood value, and the population genetic structure of the model was plotted.

## 3 Results

### 3.1 Phenotypic Traits of *Toxicodendron vernicifluum*

#### 3.1.1 Morphological Characteristics

**FIGURE 3:**
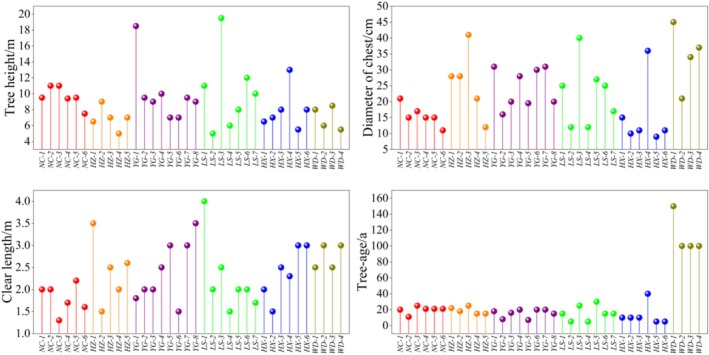
Morphological characteristics of different seed sources of *Toxicodendron vernicifluum* . Among them, NC‐1–NC‐6 is the ZNC population, HZ‐1–HZ‐5 is the ZHZ group, YG‐1–YG‐8 is the ZYG group, LS‐1–LS‐7 is the NLS group, HX‐1–HX‐6 is the NHX group, and WD‐1–WD‐4 is the DWD group.

Tree height, diameter of chest, clear length, and tree age were measured for 36 *T. vernicifluum* family lines from six populations (Figure 3). LS‐3, YG‐1, and HX‐4 ranked the top three in terms of tree height compared to other families, reaching 19.5, 18.5, and 13 m, respectively, while ZHZ and DWD *T. vernicifluum* populations had the smallest average heights, 6.9 and 7 m, respectively; WD‐1 was the thickest, with a diameter of chest of 45 cm, and HX‐5 was the smallest, with a diameter of chest of only 9 cm; in terms of the clear length, LS‐1 had the highest clear length of 4 m, followed by HZ‐1 with 3.5 m, and NC‐3 had the smallest clear length at 1.3 m. The DWD *T. vernicifluum* group was the oldest, with an average age of more than 100 years, while the rest of the family lines were aged between 5 and 40 years. Overall, the three groups ZHZ, ZYG, and NLS were wider and of better quality, while the DWD group was shorter, probably due to the fact that the trees were in their old age and their physiological, biochemical, and metabolic capacities were weaker, resulting in a poorer quality of the trees.

#### 3.1.2 Seed Characteristics

**FIGURE 4:**
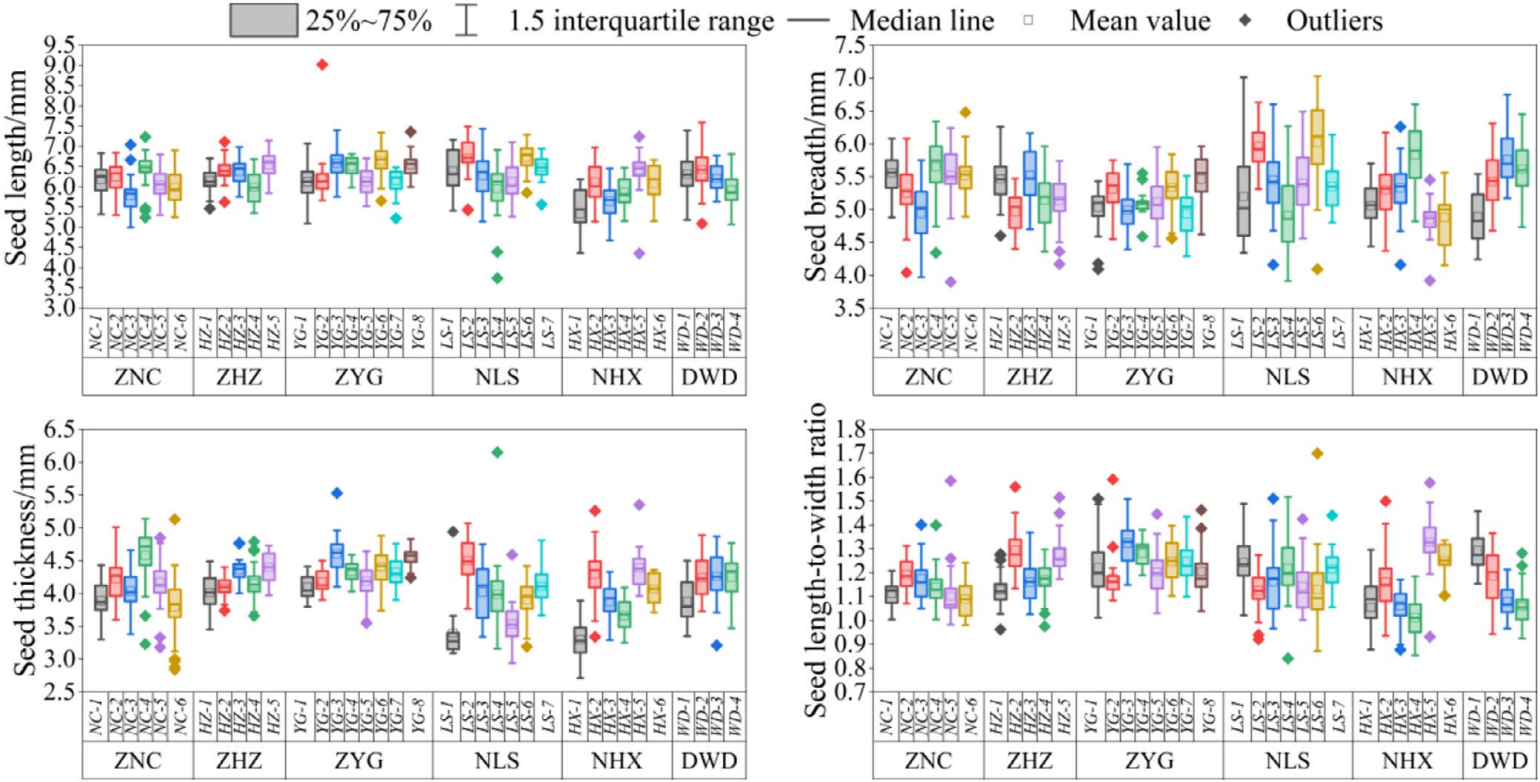
Characteristics of seed length, breadth, thickness, and seed length‐to‐width ratio of different seed sources of *Toxicodendron vernicifluum* .

The data of seed length, breadth, thickness, and seed length‐to‐width ratio of different *T. vernicifluum* family lines were more concentrated, with fewer outliers (Figure 4). The average seed length of all family lines ranged from 5.0 to 7.0 mm; the average seed breadth ranged from 4.5 to 6.5 mm; the average seed thickness ranged from 3.0 to 5.0 mm; and the average seed length‐to‐width ratio ranged from 1.0 to 1.4. The seeds of LS‐6 were the longest (6.71 mm) and broadest (6.02 mm), and the seeds of YG‐3 were the thickest (4.60 mm); the seeds of HX‐1 were the shortest and thinnest, 5.33 and 3.30 mm, respectively, and the seeds of HX‐5 were the narrowest (4.84 mm); the seeds of HX‐5 had the largest length‐to‐width ratio (1.33), and those of HX‐4 had the smallest length‐to‐width ratio (1.01). Overall, the NLS group had the longest and broadest seeds, and the ZYG group had the thickest seeds and the largest seed length‐to‐width ratio.

The coefficient of variation is an important indicator of the stability of seed traits. The higher the coefficient of variation, the higher the degree of dispersion of the trait, and accordingly, the lower the stability of the trait index. The plasticity index of the plant is a measure of its ability to cope with environmental stresses, and the higher index indicates that the plant has a stronger ability to self‐regulate and adapt to environmental changes. The coefficients of variation for seed length of *T. vernicifluum* ranged from 3.97% (YG‐4) to 18.13% (HX‐1), with plasticity indices ranging from 0.12 (YG‐4) to 0.86 (HX‐1) (Table 2). For seed breadth, the coefficients of variation ranged from 3.08% (YG‐4) to 13.72% (LS‐1), with plasticity indices ranging from 0.17 (YG‐4) to 0.42 (LS‐6). Seed thickness coefficients of variation ranged from 2.88% (YG‐8) to 13.49% (NC‐6), with plasticity indices ranging from 0.12 (YG‐4 and YG‐8) to 0.49 (LS‐4). Seed length‐to‐width ratio coefficients of variation ranged from 4.15% (YG‐4) to 17.91% (HX‐1), and the plasticity index was in the range of 0.14 (YG‐4) to 0.85 (HX‐1) (Table 2). The above results indicated that YG‐4 had the weakest plasticity of various subtrait indices but had strong stability. Overall, the coefficients of variation and plasticity indices of seed length, breadth, thickness, and seed length‐to‐width ratios of *T. vernicifluum* varied greatly, with seed length having the greatest degree of variation and the strongest plasticity, and seed thickness having the least degree of variation and the weakest plasticity.

**TABLE 2:** Coefficients of variation and plasticity indices of the length, breadth, thickness, and seed length‐to‐width ratios of *Toxicodendron vernicifluum* seeds from different seed sources.

	Seed length		Seed breadth		Seed thickness		Seed length‐to‐width ratio	
	CV%	PI	CV%	PI	CV%	PI	CV%	PI
NC‐1	5.76	0.22	4.98	0.20	6.58	0.26	4.72	0.17
NC‐2	6.52	0.23	7.48	0.34	7.89	0.28	5.69	0.18
NC‐3	7.62	0.29	9.46	0.31	6.93	0.27	7.80	0.25
NC‐4	6.75	0.28	7.39	0.32	9.01	0.37	6.69	0.29
NC‐5	6.64	0.22	8.31	0.38	8.99	0.34	10.09	0.38
NC‐6	7.17	0.24	6.11	0.25	13.49	0.45	6.66	0.21
HZ‐1	4.54	0.19	6.51	0.27	6.99	0.23	6.28	0.25
HZ‐2	4.68	0.21	5.85	0.20	3.76	0.15	7.21	0.28
HZ‐3	4.59	0.18	6.61	0.24	4.03	0.16	7.14	0.25
HZ‐4	6.38	0.20	7.73	0.27	6.41	0.24	6.15	0.25
HZ‐5	4.67	0.18	7.76	0.27	4.90	0.16	6.32	0.23
YG‐1	7.08	0.28	6.22	0.25	4.21	0.14	9.54	0.33
YG‐2	9.30	0.37	5.61	0.21	3.94	0.13	7.78	0.32
YG‐3	5.69	0.22	6.12	0.23	6.10	0.26	7.09	0.24
YG‐4	3.97	0.12	3.08	0.17	3.56	0.12	4.15	0.14
YG‐5	5.51	0.18	7.13	0.25	6.04	0.23	8.15	0.29
YG‐6	6.03	0.23	6.30	0.22	6.06	0.23	6.33	0.21
YG‐7	4.72	0.19	6.90	0.22	5.25	0.18	6.79	0.23
YG‐8	4.50	0.19	6.99	0.22	2.88	0.12	7.80	0.29
LS‐1	8.48	0.24	13.72	0.38	13.12	0.37	9.72	0.32
LS‐2	7.18	0.28	5.37	0.20	7.46	0.26	7.40	0.28
LS‐3	7.95	0.31	9.87	0.37	10.58	0.30	11.92	0.36
LS‐4	10.87	0.46	11.39	0.38	13.18	0.49	10.79	0.45
LS‐5	6.69	0.26	9.14	0.30	8.93	0.36	9.64	0.30
LS‐6	4.92	0.20	10.68	0.42	7.19	0.28	12.97	0.49
LS‐7	4.56	0.20	6.12	0.23	6.31	0.24	6.73	0.26
HX‐1	18.13	0.86	6.43	0.22	8.85	0.30	17.91	0.85
HX‐2	7.56	0.23	7.89	0.29	10.20	0.37	10.01	0.37
HX‐3	7.32	0.28	7.46	0.34	7.11	0.24	7.16	0.25
HX‐4	6.10	0.20	8.11	0.27	6.56	0.21	8.38	0.28
HX‐5	7.38	0.40	5.31	0.28	6.39	0.26	8.05	0.41
HX‐6	8.22	0.23	9.44	0.25	5.49	0.15	6.31	0.18
WD‐1	7.41	0.30	7.85	0.23	8.43	0.26	6.09	0.21
WD‐2	8.67	0.33	7.36	0.26	7.27	0.24	8.86	0.31
WD‐3	5.52	0.17	6.90	0.23	7.98	0.34	5.62	0.20
WD‐4	6.63	0.26	7.58	0.27	7.61	0.27	7.58	0.28

Abbreviations: CV%, coefficient of variation; PI, plasticity index.

#### 3.1.3 Phenotypic Trait Correlation Analysis

**FIGURE 5:**
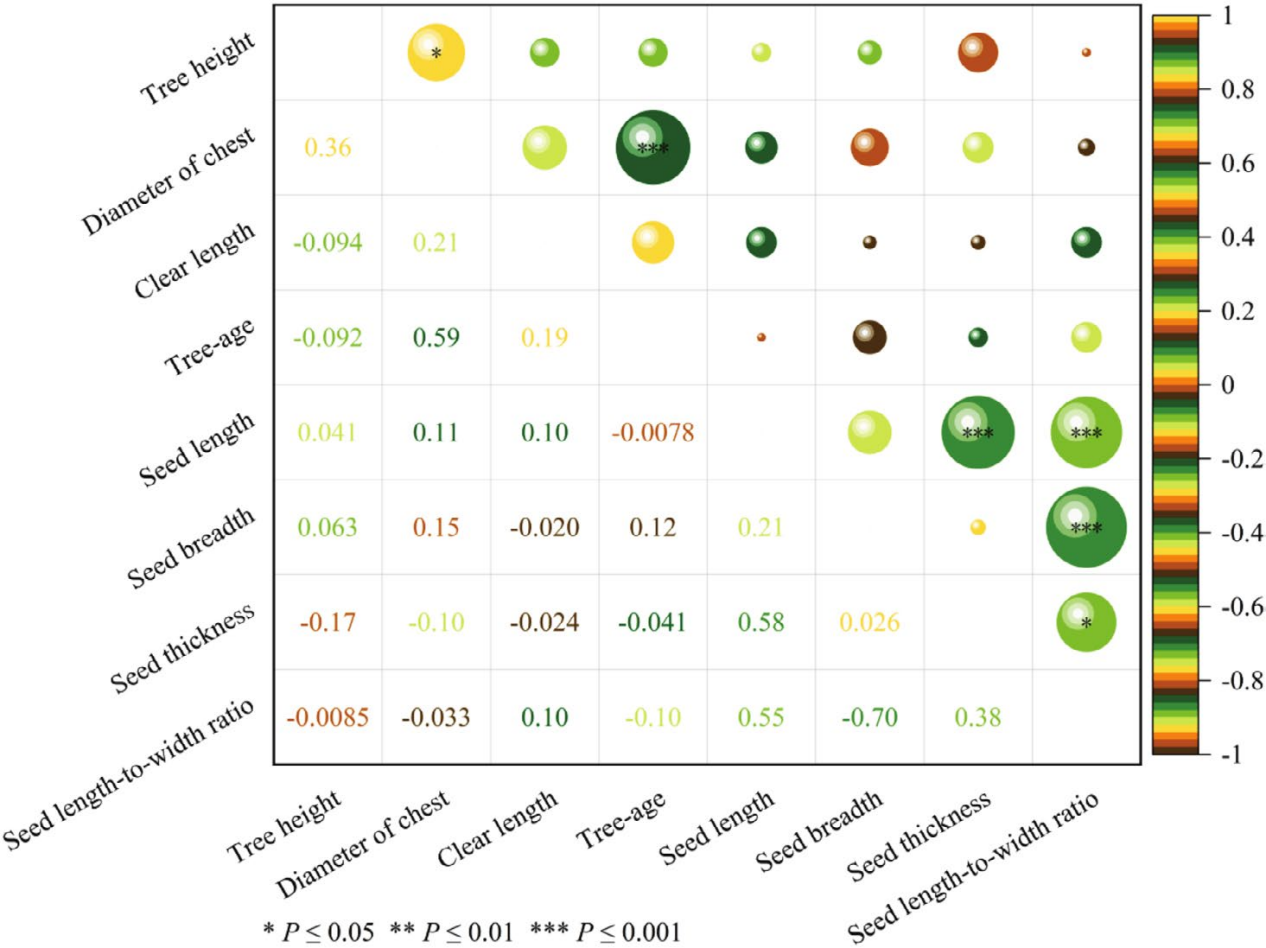
Correlation analysis of phenotypic traits in *Toxicodendron vernicifluum* .

There was a highly significant positive correlation between diameter of chest and tree age at the 0.001 level (*p* ≤ 0.001) (Figure 5). Seed length to seed thickness and seed length to seed length‐to‐width ratio had highly significant positive correlations at the 0.01 level (*p* ≤ 0.01), while seed breadth had a highly significant negative correlation with seed length‐to‐width ratio at the 0.01 level (*p* ≤ 0.01). A significant positive correlation (*p* ≤ 0.05) was found between tree height and diameter of chest, seed thickness, and seed length‐to‐width ratio.

#### 3.1.4 Principal Component Analysis of Phenotypic Traits

**FIGURE 6:**
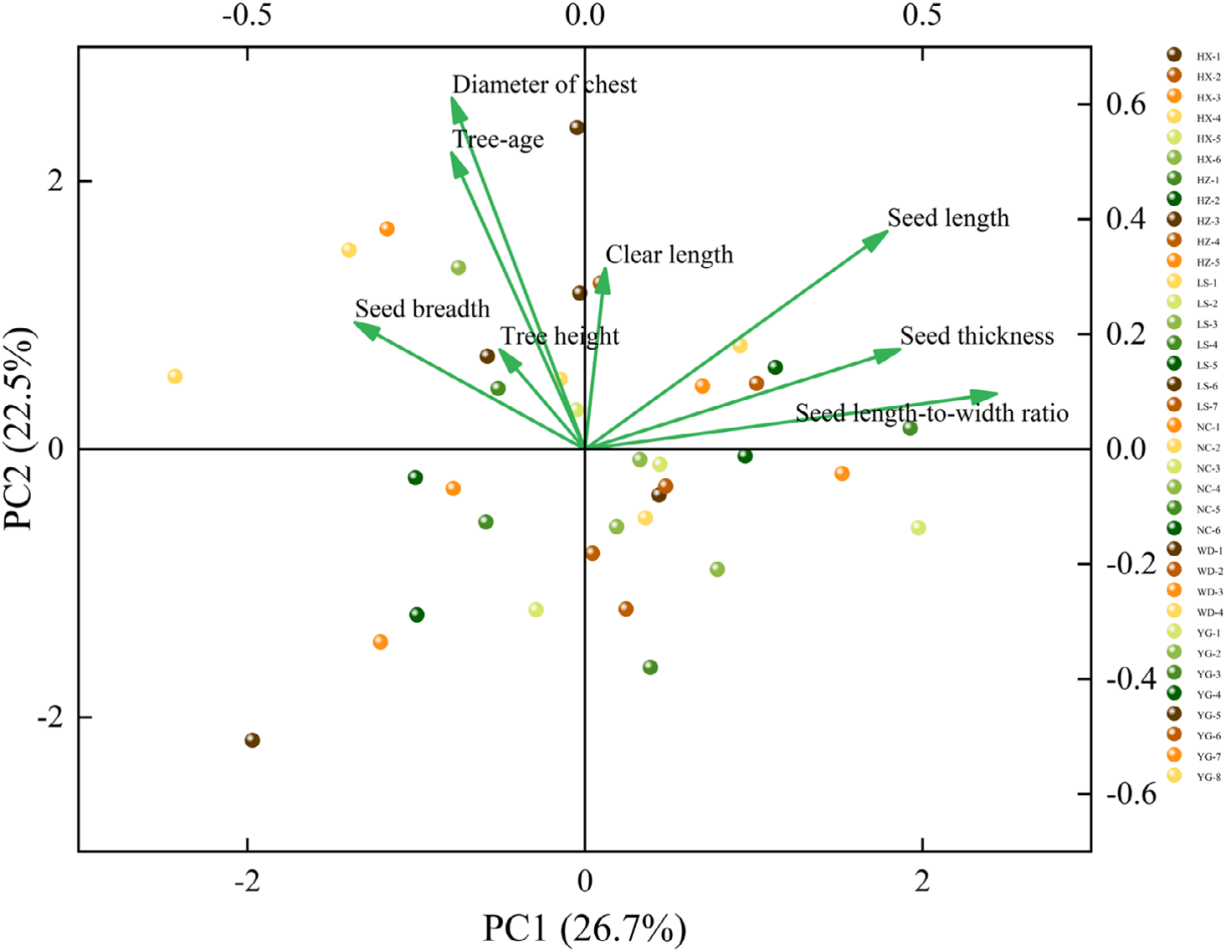
Principal component analysis of phenotypic traits in *Toxicodendron vernicifluum* .

As shown in Figure 6, the principal component relationships of the morphological and seed traits of *T. vernicifluum* from different seed sources behaved differently. Two principal axes of trait variation were identified, in which the variance explained rate of PC1 and PC2 axes was 26.7% and 22.5%, respectively, totaling 49.2%. The PC1 axis was mainly related to seed thickness and seed length‐to‐width ratio, while the PC2 axis was mainly related to the diameter of the chest and clear length. In conclusion, the phenotypic traits were closely related to each other, and seed thickness, seed length‐to‐width ratio, diameter of the chest, and clear length were the main components of different seed sources of *T. vernicifluum* , which could represent most of the information.

### 3.2 Screening of SSR Core Primers for *Toxicodendron vernicifluum*

#### 3.2.1 Concentration and Purity of DNA

The DNA concentration of the 36 *T. vernicifluum* families ranged from 63.7 ng/μL (LS‐6) to 2985.3 ng/uL (WD‐1). All family DNAs showed a significant absorption peak at OD260, and the OD260/OD280 ratios were basically between 1.7 and 1.9 (Table 3). The results indicated that the concentration and purity of *T. vernicifluum* DNA extracted by the kit were good and could be used for subsequent screening of SSR primers and SSR molecular labeling tests.

**TABLE 3:** Concentration and purity of genomic DNA of *Toxicodendron vernicifluum* .

Genealogy	Concentration/(ng μL^−1^)	OD_260_/OD_280_	Genealogy	Concentration/(ng μL^−1^)	OD_260_/OD_280_	Genealogy	Concentration/(ng μL^−1^)	OD_260_/OD_280_
NC‐1	274.5	1.862	YG‐2	410.6	1.763	LS‐6	63.7	1.848
NC‐2	690.4	1.782	YG‐3	1887.0	1.724	LS‐7	1256.0	1.904
NC‐3	432.7	1.667	YG‐4	605.0	1.746	HX‐1	752.6	1.721
NC‐4	786.4	1.956	YG‐5	1787.2	1.803	HX‐2	845.0	1.833
NC‐5	346.6	1.667	YG‐6	433.6	1.738	HX‐3	1298.8	1.889
NC‐6	700.1	1.707	YG‐7	476.7	1.762	HX‐4	433.8	1.812
HZ‐1	698.7	1.892	YG‐8	494.6	1.768	HX‐5	214.1	1.788
HZ‐2	511.9	1.664	LS‐1	1062.1	1.868	HX‐6	731.3	1.923
HZ‐3	1054.1	1.837	LS‐2	390.4	1.832	WD‐1	2985.3	1.830
HZ‐4	1511.7	1.829	LS‐3	882.1	1.894	WD‐2	1209.6	1.812
HZ‐5	141.3	1.801	LS‐4	1068.5	1.857	WD‐3	609.3	1.844
YG‐1	722.4	1.706	LS‐5	637.6	1.786	WD‐4	1051.9	1.820

#### 3.2.2 Core Primer Screening Results

**FIGURE 7:**
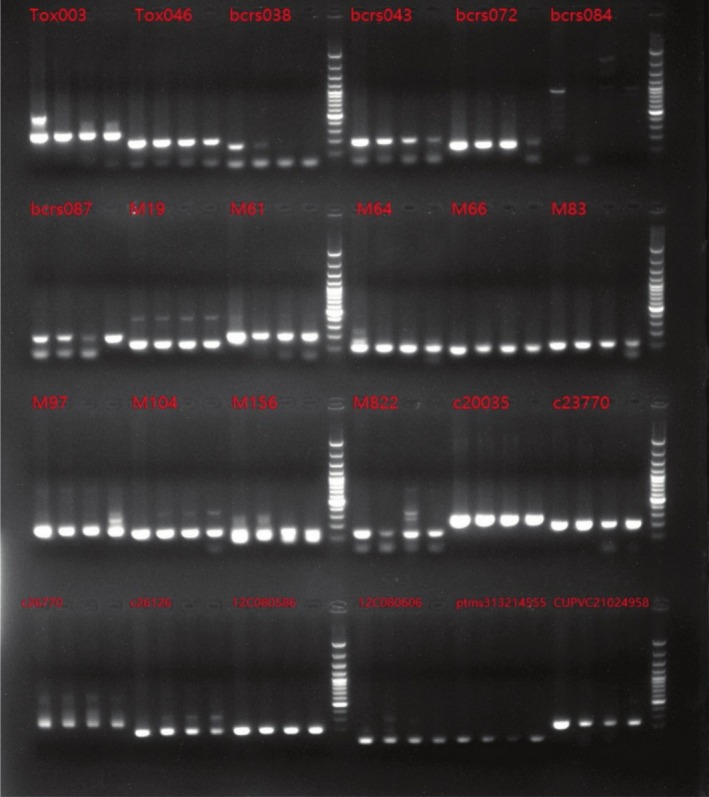
Amplification effect of 24 pairs of SSR primers.

**FIGURE 8:**
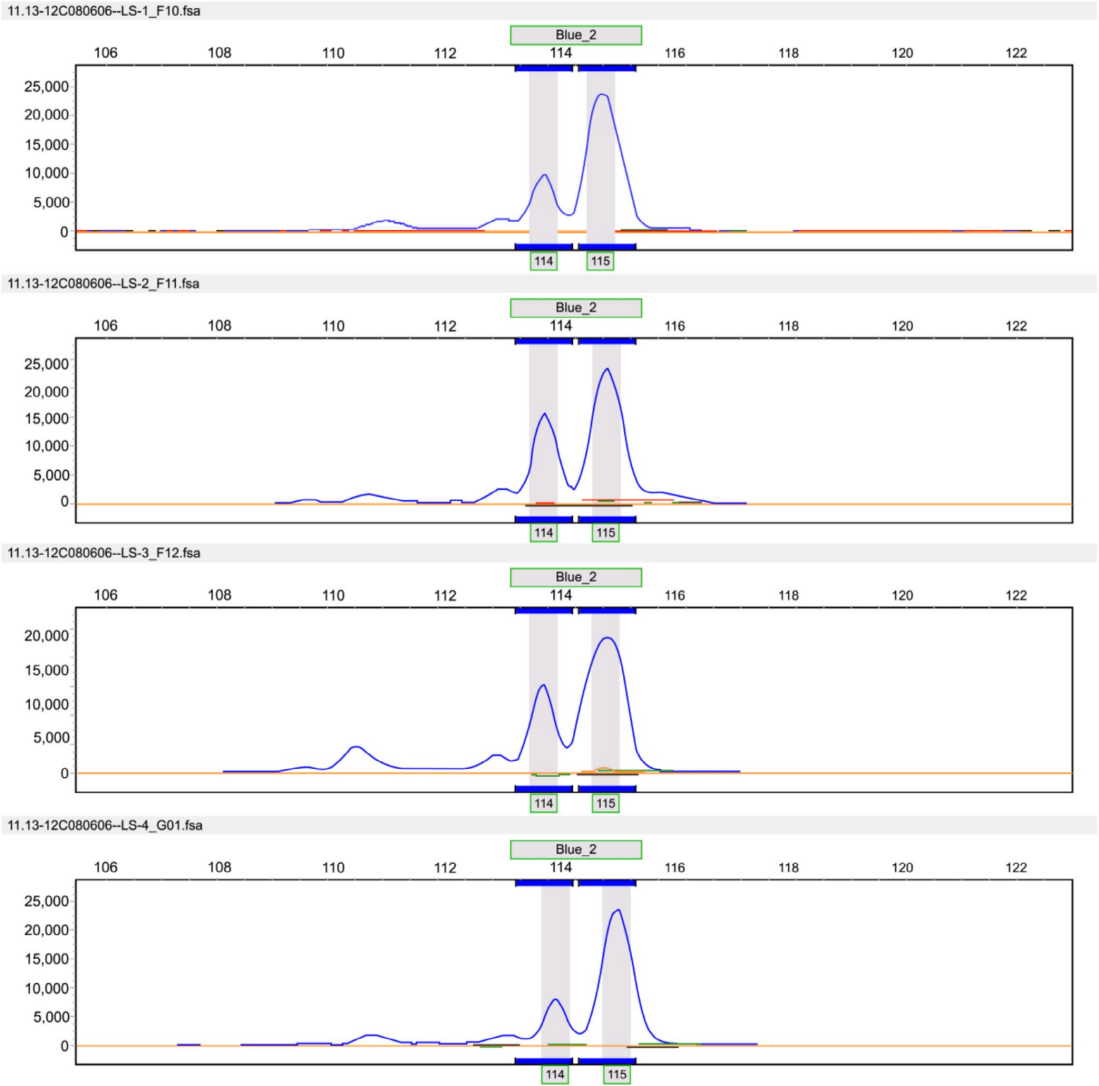
Capillary electrophoresis profiles of some *Toxicodendron vernicifluum* SSR‐PCR amplification results.

From the initial screening and rescreening of 160 primer pairs, 24 pairs of SSR primers with clear bands, high polymorphism, and good reproducibility suitable for different germplasms of *T. vernicifluum* in Yunnan, China, were obtained. The forward sequence, reverse sequence, fragment size, and annealing temperature of the primers are shown in Table 4. The fragment sizes are mainly concentrated between 100 and 200 bp, and the annealing temperatures are concentrated between 50°C and 60°C. On this basis, four DNA samples from different *T. vernicifluum* families were randomly selected for PCR amplification with each pair of primers. Agarose gel electrophoresis was then performed, and the results were detected with a UV gel imaging system to obtain amplification effect diagrams of the primers (Figure 7), which further verified the accuracy of the selection to meet the requirements for subsequent SSR marker analysis. After using these primers to perform SSR amplification and obtaining stable and highly polymorphic amplification results, SSR molecular marker experiments were carried out. Figure 8 shows the capillary electrophoresis profiles of the SSR‐PCR amplification results for some *T. vernicifluum* samples.

**TABLE 4:** Information related to 24 pairs of SSR primers.

Primer no.	Primer sequence (5′‐3′)		Fragment size/bp	Annealing temperature/°C
	Forward	Reverse		
bcrs038	TTTTGGCGTTTTCTCCTAATAGTC	GAGTATATAATCATGAGAGGGAAAG	150–180	50.5
bcrs043	CTCTTATTCCTTTGAACTGAAAACG	GTGCAGACTTTCGTTATTTATAGTCG	191–231	52.5
bcrs072	GGCTCTCTTGCTTACTGCATC	CTAAACGATCACATTAGAGGGAAATTA	150–170	53.1
bcrs087	AAATGTATGAAGACAAGCCTCACA	TGACCTTTATAGGGCATGAATCTT	150–170	54.0
CUPVC21024958	GCGTTTGTTGCCTTTTCTTC	GTGAACCCCGTCTCAATGTC	245–265	54.5
c20035	TGCTGGAGGATTATTAGCCG	CATTTGGTGGCCAGTTCATA	275–289	53.5
c23770	TTCTGATCCCAAGAACCCAG	GGGATTGATGGAAAGGGAAT	220–236	53.1
c26126	ATTCTCTGGTGGAGGTGGTG	GCAATCAGCCATCAGAACAA	150–200	55.5
c26770	TCCGCCTCCAATAACTGAAC	GCTTCTCAAGGGGCTTCTCT	202–243	56.0
M19	AGTGAATAGGTAGAATTCTCC	CGGATTTTAGCTCAATTCCATC	110–130	50.0
M61	CCGTTCACTGATTTTGCTAG	CTGGCTACTAGATGATCCAG	169–207	51.3
M64	ATAGTGAGTGCATGGTGGCG	CTCCTCTTGAAACTGAGCTG	100–120	55.0
M66	TGGAGCACTCATTTGTAACG	CTGGATCTATACTCAATTCC	90–120	50.0
M83	CATTCAACGCCGACAATTCC	TCCATATTCAGCCCAAGTGC	115–126	55.0
M97	AGTTCTGGAGCTCAACATGG	TCGAAGCTCTGATACCACTG	163–179	54.0
M104	TGGATTAGGCGAGTCACACC	GTTTCACAGCATCCACGTGC	149–157	57.5
M156	AAGCTAGCAAATACACATAGG	CTGACAAGTTCCAGACAGGG	40–152	52.0
M822	GGTGGATTGAAGAAATGACG	AAATTCATTCGCTTTCACCTC	127–149	50.8
ptms313214555	TGATGAACAAGTCCAAAAGGG	AAAACAGCACAGCATGCATC	112–145	53.5
Tox003	AGACAATGGGATTTTTCCCC	CGATTTGAGGCGGTGTATTT	250–280	52.6
Tox046	CGTCCCAAACTTAATTTGATATACG	CAGTGGCAGCAAGAATTGAA	200–210	52.3
YFMS‐77	ATGTCACATGCAACAGAGGC	GAGATGAGTATGCTCTCGGCTT	120–130	56.2
12C080586	CTGCCTTCCTTGGATGTGGT	GCCGTTGCTCTGATGAT	188–205	55.0
12C080606	GCTGGATAGTAGACAGGGACAG	GGGTAAACGGCGGGAGTA	100–120	57.2

### 3.3 SSR‐Based Genetic Diversity Analysis of *Toxicodendron vernicifluum*

#### 3.3.1 Genetic Diversity Parameters for 24 Loci

Table 5 shows the genetic diversity parameters at 24 loci. About 44 alleles (Na) were detected in 36 *T. vernicifluum* family lines, varying between 1 (bcrs072) and 5.5 (M156) with a mean of 1.826. The number of effective alleles (Ne) ranged from 1.000 (bcrs072) to 4.242 (M156) with a mean of 1.517. The Shannon information index (*I*) varied between 0.000 (bcrs072) and 1.508 (M156) with a mean value of 0.377. Expected heterozygosity (He) and observed heterozygosity (Ho) ranged between 0.000 (bcrs072) ~ 0.736 (M156) and 0.000 (bcrs072) ~ 1.000 (M66), with mean values of 0.230 and 0.289, respectively. The Ho of loci bcrs043, c20035, c23770, c26770, M19, M64, M66, M83, M97, M104, M156, M822, Tox003, Tox046, and 12C080606 are all greater than He, indicating that all 15 loci have relatively high heterozygosity. The fixation index (*F*) varied from −0.897 (M66) to 0.444 (12C080586), with a mean value of −0.208. The polymorphic information coefficients (PIC) of the 24 loci ranged from 0.000 (bcrs072) to 0.829 (M156), with an average of 0.257 and a mean higher than 0.250. The PIC of two of the loci (M156 and Tox003) had PIC as high as 0.829 and 0.784, respectively, and the PIC of all loci were mostly higher than 0.250, showing high polymorphism. The gene flow (Nm) among *T. vernicifluum* families ranged from 0.000 (bcrs072) to 8.700 (M66). The inbreeding coefficient (Fit) within the total population ranged from −0.810 (M66) to 1.000 (bcrs072), with an average of 0.134. The coefficient of population differentiation (F_st_) varied from 0.028 (M66) to 1.000 (bcrs072) with an average value of 0.220. The *F* statistic can reflect the genetic differentiation status among populations, among which the inbreeding coefficient (Fis) within a population is a measure of the degree of deviation from Hardy–Weinberg equilibrium within a population. When Fis > 0, there is inbreeding and insufficient heterozygosity within a population, and vice versa, heterozygous mating and excessive heterozygosity. The inbreeding coefficient (Fis) within the populations ranged from −0.862 (M66) to 0.498 (12C080586), with an average of −0.103, indicating heterozygous mating and excessive heterozygosity in *T. vernicifluum* germplasm resources.

**TABLE 5:** Genetic diversity parameters of 24 loci in *Toxicodendron vernicifluum* .

Locus	Na	Ne	I	Ho	He	F	PIC	Fis	Fit	Fst	Nm
bcrs038	1.500	1.230	0.181	0.067	0.097	0.310	0.128	0.310	0.476	0.240	0.791
bcrs043	1.667	1.408	0.331	0.222	0.208	−0.125	0.295	−0.070	0.443	0.479	0.272
bcrs072	1.000	1.000	0.000	0.000	0.000	0.000	0.000	N/A	1.000	1.000	0.000
bcrs087	1.667	1.285	0.292	0.133	0.176	0.293	0.179	0.244	0.336	0.122	1.808
CUPVC21024958	1.500	1.276	0.380	0.233	0.257	0.146	0.319	0.092	0.612	0.572	0.187
c20035	1.833	1.648	0.521	0.567	0.362	−0.547	0.323	−0.568	−0.396	0.109	2.036
c23770	2.000	1.403	0.398	0.242	0.237	−0.033	0.272	−0.021	0.124	0.143	1.503
c26126	2.167	1.629	0.559	0.311	0.349	0.180	0.515	0.108	0.465	0.400	0.376
c26770	1.167	1.047	0.063	0.042	0.036	−0.143	0.042	−0.143	−0.021	0.106	2.100
M19	2.000	1.774	0.604	0.566	0.420	−0.330	0.359	−0.349	−0.193	0.116	1.911
M61	1.833	1.356	0.364	0.224	0.229	−0.040	0.215	0.023	0.139	0.118	1.861
M64	1.167	1.030	0.048	0.028	0.025	−0.091	0.029	−0.091	−0.014	0.070	3.300
M66	2.333	2.267	0.799	1.000	0.537	−0.897	0.439	−0.862	−0.810	0.028	8.700
M83	1.167	1.022	0.039	0.021	0.020	−0.067	0.031	−0.067	−0.011	0.053	4.500
M97	2.333	2.183	0.791	0.967	0.535	−0.834	0.561	−0.807	−0.528	0.155	1.368
M104	1.167	1.047	0.063	0.042	0.036	−0.143	0.046	−0.143	−0.021	0.106	2.100
M156	5.500	4.242	1.508	0.935	0.736	−0.277	0.829	−0.270	−0.104	0.131	1.659
M822	1.167	1.100	0.094	0.083	0.063	−0.333	0.046	−0.333	−0.043	0.217	0.900
ptms313214555	1.333	1.069	0.094	0.028	0.049	0.429	0.076	0.429	0.489	0.106	2.100
Tox003	3.167	2.490	0.992	0.591	0.583	−0.031	0.784	−0.014	0.275	0.285	0.626
Tox046	1.500	1.344	0.291	0.292	0.198	−0.433	0.262	−0.469	0.120	0.401	0.374
YFMS‐77	1.333	1.079	0.100	0.042	0.053	0.220	0.088	0.220	0.317	0.125	1.757
12C080586	1.333	1.083	0.117	0.033	0.066	0.444	0.079	0.498	0.543	0.088	2.586
12C080606	2.000	1.399	0.408	0.274	0.251	−0.101	0.258	−0.093	0.029	0.111	1.995
Mean	1.826	1.517	0.377	0.289	0.230	−0.208	0.257	−0.103	0.134	0.220	1.867

#### 3.3.2 Genetic Diversity Parameters of the Six Populations

The genetic diversity parameters of six populations of *T. vernicifluum* from Niuchang Town, Haizi Town, and Yigu Town in Zhaotong City, Yunnan Province, China; Luzhang Town and Hexi Township in Nujiang Prefecture, Yunnan Province, China; and Weideng Township in Diqing Prefecture, Yunnan Province, China, based on SSR markers are shown in Table 6. The total Na for the six groups is approximately 11, ranging from 1.542 (DWD) to 2.417 (ZYG), with an average of 1.826; Ne was 1.359 (NLS) to 1.755 (ZYG) with an average of 1.517; *I* was 0.277 (NLS) to 0.553 (ZYG) with an average of 0.377; Ho was 0.214 (NLS) to 0.341 (ZYG) with an average of 0.289; He was 0.170 (NLS) to 0.320 (ZYG) with an average of 0.230; and *F* was −0.473 (DWD) to −0.007 (ZYG) with an average of −0.241. The data showed that the genetic diversity of the six *T. vernicifluum* populations varied, and based on *I* and He, the ZYG population had the highest genetic diversity, with *I* and He of 0.553 and 0.320, respectively, and the NLS population had the lowest genetic diversity, with *I* and He of 0.277 and 0.170, respectively. The relative sizes of Ho and He could reflect the mating characteristics and heterozygous status of the individuals in the populations. The relative size of Ho and He can reflect the mating characteristics and heterozygous status of individuals within a population. When Ho < He, there is inbreeding within the population and the population lacks heterozygosity, and vice versa; it indicates that the population is heterozygous and there is an excess of heterozygosity. The mean value of Ho for the six populations studied was 0.289, and the mean value of He was 0.230, with Ho > He. Overall, the *T. vernicifluum* populations showed an excess of heterozygotes, indicating that there was no inbreeding and lack of heterozygosity in any of the six populations.

**TABLE 6:** Genetic diversity parameters of six populations of *Toxicodendron vernicifluum* .

Populations	Na	Ne	I	Ho	He	F
ZNC	1.750	1.486	0.364	0.282	0.220	−0.267
ZHZ	2.000	1.677	0.441	0.297	0.264	−0.134
ZYG	2.417	1.755	0.553	0.341	0.320	−0.007
NLS	1.625	1.359	0.277	0.214	0.170	−0.125
NHX	1.625	1.456	0.338	0.319	0.220	−0.439
DWD	1.542	1.370	0.287	0.281	0.187	−0.473
Mean	1.826	1.517	0.377	0.289	0.230	−0.241

*Note:* ZNC: Niuchang Town, Zhenxiong County, Zhaotong City, Yunnan Province, China; ZHZ: Haizi Town, Yiliang County, Zhaotong City, Yunnan Province, China; ZYG: Yigu Town, Zhenxiong County, Zhaotong City, Yunnan Province, China; NLS: Luzhang Town, Lushui City, Nujiang Prefecture, Yunnan Province, China; NHX: Hexi Township, Lamping County, Nujiang Prefecture, Yunnan Province, China; DWD: Wideng Township, Wisi County, Diqing Prefecture, Yunnan Province, China.

#### 3.3.3 Molecular Analysis of Variance (AMOVA) for Genetic Differentiation in Six Populations

AMOVA analysis of six populations of *T. vernicifluum* revealed that 11% of the total genetic variation was due to differences among populations, 47% was attributed to variation among individuals within populations, and 42% originated from within individuals (Table 7). This indicates that the majority of the genetic variation exists within populations, with a substantial proportion also found within individuals.

**TABLE 7:** Analysis of molecular variance (AMOVA) for genetic differentiation of six populations of *Toxicodendron vernicifluum* .

Source of variation	Degree of freedom	Sum of squares	Mean squares	Estimated variance	Percent of total variation
Among populations	5	80.772	16.154	0.652	11%
Within populations	30	251.950	8.398	2.914	47%
Within the individual	36	92.500	2.569	2.569	42%
Total	71	425.222		6.136	100%

### 3.4 Analysis of the Genetic Structure of *Toxicodendron vernicifluum* Populations

#### 3.4.1 Genetic Structure

**FIGURE 9:**
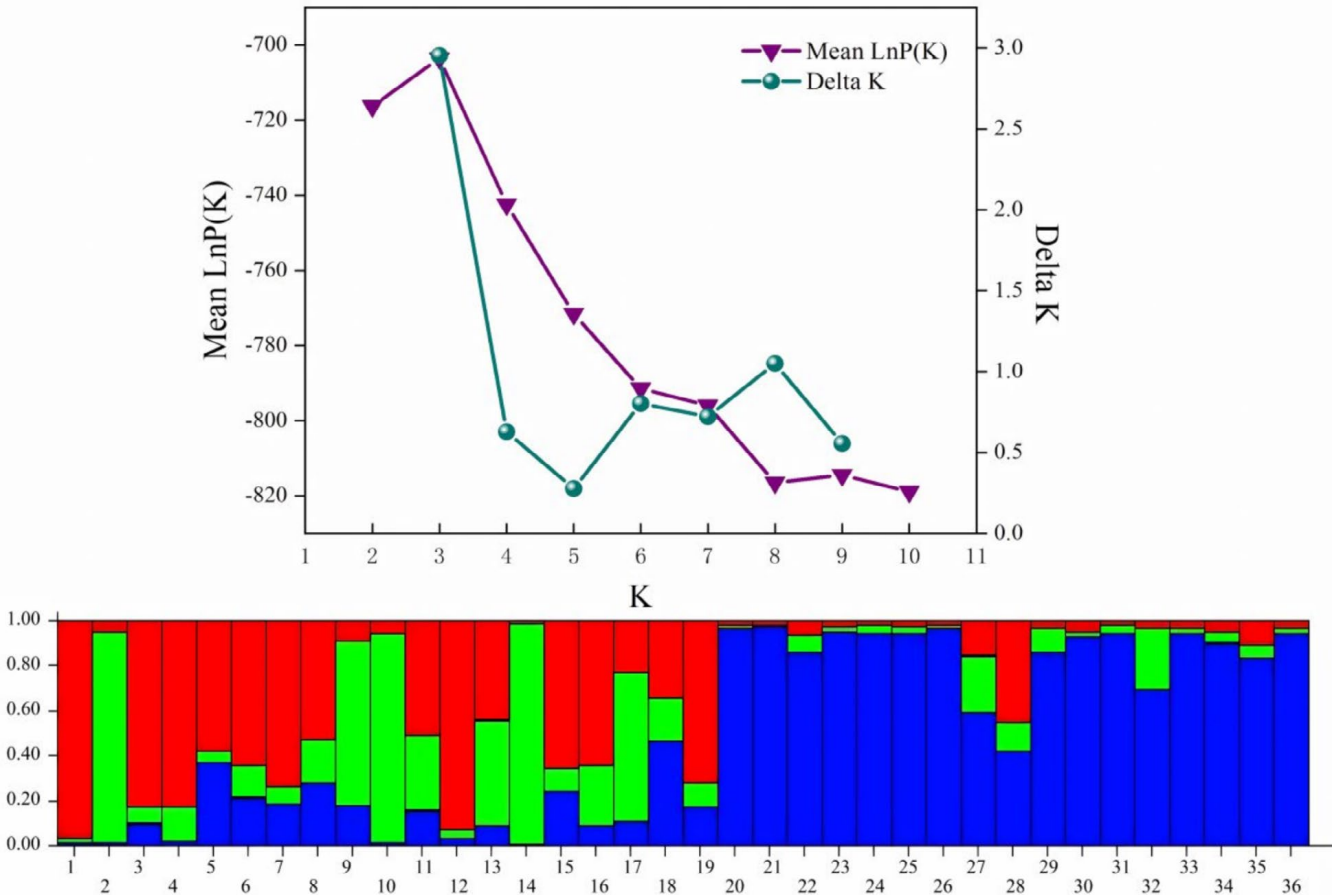
Analysis of the genetic structure of the *Toxicodendron vernicifluum* germplasm resource, where 1–6 represents NC‐1–NC‐6 (ZNC), 7–11 represents HZ‐1–HZ‐5 (ZHZ), 12–19 represents YG‐1–YG‐8 (ZYG), 20–26 represents LS‐1–LS‐7 (NLS), 27–32 represents HX‐1–HX‐6 (NHX), and 33–36 represents WD‐1–WD‐4 (DWD) populations.

The results of the STRUCTURE analysis showed that the ΔK value was the largest when K = 3, indicating that the natural populations of Yunnan *T. vernicifluum* should be divided into three subgroups (Figure 9). According to the principle of maximum likelihood, the 36 *T. vernicifluum* germplasm resources can also be divided into three taxa according to genetic components, of which the red part is the 1st taxon, mainly composed of ZNC, ZHZ, and ZYG populations; the green part is the 2nd taxon, mainly composed of NC‐2, ZHZ, and ZYG groups; and the blue part is the 3rd taxon, mainly composed of NLS, NHX, and DWD groups. From the figure, we can also see that the genetic backgrounds of all *T. vernicifluum* germplasm resources are not single, all belong to two or three taxa, and the genetic backgrounds are more complex. The genetic backgrounds of the germplasm resources of the ZNC, ZHZ, and ZYG groups basically belong to taxa 1 and 2, and the proportions of these two taxa are roughly the same, and the proportion of those belonging to taxa 3 is very small. The genetic backgrounds of the NLS, NHX, and DWD groups basically belong to the other taxa, and only a small part of them belong to other groups. In addition, the genetic components of different germplasm from the same group are basically the same.

#### 3.4.2 Cluster Analysis and PCoA Analysis

**FIGURE 10:**
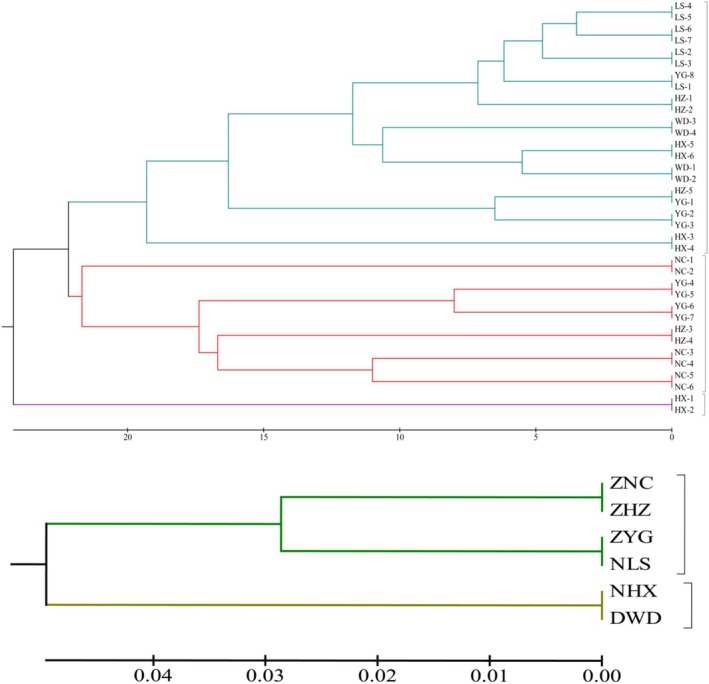
Cluster analysis of *Toxicodendron vernicifluum* resources based on SSR markers.

Individuals and populations of *T. vernicifluum* were analyzed by UPGMA clustering based on genetic distance (Figure 10). The results showed that different individuals of the same population were basically clustered together, indicating that the genetic similarity among individuals within the population was relatively low. The 36 *T. vernicifluum* families were divided into three clusters; families HX‐1 and HX‐2 were clustered into one group, and the NLS population (LS‐1 ~ LS‐7), DWD population (WD‐1 ~ WD‐4), HZ‐1, HZ‐2, HZ‐5, YG‐1, YG‐2, YG‐3, YG‐8, HX‐3, HX‐4, HX‐5, and HX‐6, a total of 22 family lines, were clustered into one group, and the remaining 12 family lines were clustered into one group. Among them, the first and the third clusters of STRUCTURE have similar family composition. From Figure 10, it was also found that the NHX and DWD populations were separated from the large group formed by the other four populations first, indicating that these two populations showed distant affinities with the other materials. These two populations were geographically relatively close to each other and were all at an altitude of more than 2000 m, which was a greater differentiation from the rest of the four groups that were basically at an altitude of less than 2000 m. The remaining four groups were further divided into two subclusters, ZNC and ZHZ into one group, and ZYG and NLS into one group. In terms of altitudinal environments, Niuchang Town, Zhenxiong County, Yunnan Province, and Haizi Town, Yiliang County, Yunnan Province, have roughly the same altitude, and Yigu Town, Zhenxiong County, Yunnan Province, and Luzhang Town, Lushui City, Yunnan Province, have roughly the same elevation, which indicates that similar climatic environments for survival might be the reason why different populations of *T. vernicifluum* form the same clusters.

**FIGURE 11:**
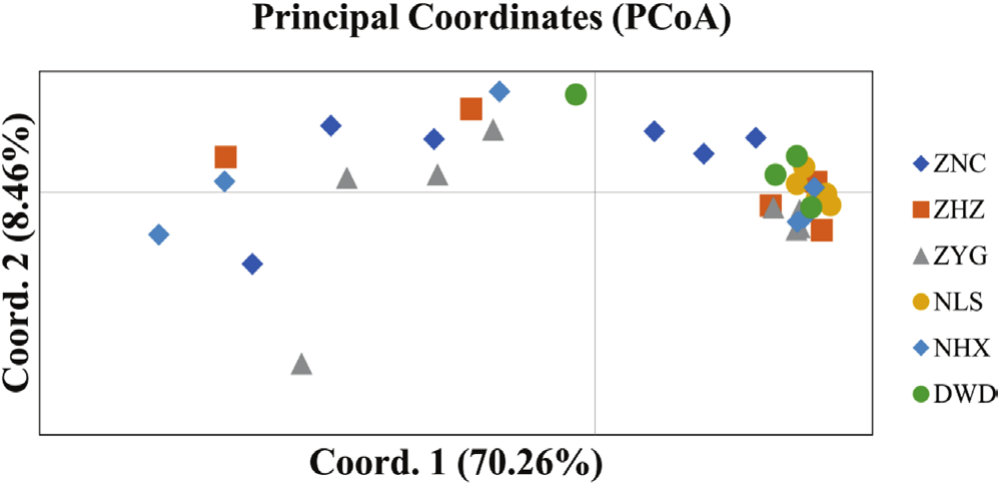
Principal coordinates analysis of 36 *Toxicodendron vernicifluum* germplasm resources within six populations.

Principal coordinate analysis (PCoA) effectively presents the spatial distance relationship between groups and individuals by presenting the coordinates in a visual way, thus intuitively revealing the similarities or differences between them. PCoA analysis of 36 *T. vernicifluum* germplasm resources was performed using GenAlex software (Figure 11). The results identified two principal axes of trait variation, with the explanatory rates of Coord. 1 and Coord. 2 being 70.26% and 8.46%, respectively, accounting for a total of 78.72%. The 36 germplasm samples are distributed in two mutually independent regions. The left region mainly contains germplasm resources from the ZNC, ZYG, and NHX populations, with a relatively scattered distribution; the right region mainly contains germplasm resources from the ZHZ, NLS, and DWD populations, with a more concentrated distribution. Among the six groups, the samples of ZNC, ZHZ, ZYG, and NHX are intertwined with each other and have high genetic similarity, while only a few samples of the DWD group are intertwined with each other, and only the NLS group is independently distributed in a separate area.

#### 3.4.3 Analysis of Genetic Distances and Affinities of Populations and Individuals of *Toxicodendron vernicifluum*

The genetic similarity coefficients and genetic distance analysis among six populations of *T. vernicifluum* are shown in Table 8. The genetic similarity coefficients among *T. vernicifluum* populations range from 0.854 to 0.967, and the genetic distances range from 0.034 to 0.158. Among them, the genetic difference between the NLS population and the NHX population is the smallest, while the genetic difference between the ZNC population and the NHX population is the largest. The genetic similarity coefficients and genetic distances of 36 *T. vernicifluum* germplasms are shown in Appendix S3. The genetic similarity coefficients among *T. vernicifluum* individuals range from 0.083 to 0.965, with an average of 0.627, and the genetic distances range from 0.036 to 2.485, with an average of 0.544. Among them, the difference between materials LS‐5 and LS‐7 is relatively small, with a smaller genetic distance, while the genetic background between YG‐3 and HX‐2 is more distant, and the difference is greater.

**TABLE 8:** Genetic similarity coefficients (on the upper diagonal) and genetic distances (on the lower diagonal) between populations of *Toxicodendron vernicifluum* .

Populations	ZNC	ZHZ	ZYG	NLS	NHX	DWD
ZNC	****	0.877	0.925	0.879	0.854	0.873
ZHZ	0.131	****	0.905	0.915	0.885	0.899
ZYG	0.078	0.099	****	0.894	0.882	0.883
NLS	0.129	0.089	0.112	****	0.967	0.964
NHX	0.158	0.122	0.126	0.034	****	0.937
DWD	0.136	0.106	0.125	0.036	0.066	****

*** means that the two groups are exactly the same and completely correlated. For example, the genetic distance or genetic similarity between “ZNC” and “ZNC” is exactly the same.

## 4 Discussion

### 4.1 Analysis of Phenotypic Traits in *Toxicodendron vernicifluum*

Under different growth environments, forest trees show different morphological characteristics. In mild climates and favorable habitats, plant bodies can accumulate more nutrients to promote their own growth; the trees are usually tall and sturdy. In cold, unfavorable habitats, the trees are usually shorter or weaker (Lv et al. 2024). In this study, the ZHZ, ZYG, and NLS populations of *T. vernicifluum* are tall and of high quality, while the DWD population is shorter and the trees are relatively older. One of the reasons may be that the average altitude of Diqing Prefecture is relatively high compared to Zhaotong and Nujiang Prefectures, and the climate is colder, which hinders the absorption and accumulation of nutrients in the plant body, making the plants of the DWD group shorter, while under the altitude conditions of a mild and suitable climate, *T. vernicifluum* can accumulate more nutrients to promote their own growth. Germplasm resources constitute a solid cornerstone of breeding work and have a profound impact on germplasm screening, innovation, and variety improvement, and other research in the field of breeding (Grzęda et al. 2023). Seed size has a significant impact on seedling growth and survival under specific growing conditions, and compared to small seeds, seedlings with large seeds are more adaptable to various pressures brought by environmental changes (Moles et al. 2007). In this study, the NLS population had the longest and widest seeds, with LS‐6 being the best performer. The ZYG population had the thickest seeds and the largest length‐width ratio, with YG‐3 having the greatest thickness. In the subtropical karst mountains of Southwest China, the more serious phenomenon of soil rock desertification, coupled with the special characteristics of the geological structure of limestone, makes the plants in this region susceptible to the stress of abiotic factors, such as acid, aluminum, and calcium salts, at the early stage of growth (Wang et al. 2021). In view of this, priority should be given to selecting and cultivating those large‐grained *T. vernicifluum* seeds that can promote plant seedling growth and increase the survival rate in this region, and the *T. vernicifluum* seeds of NLS and ZYG populations can be prioritized. The coefficient of variation of seeds is an indicator used to measure the phenotypic trait variability among different varieties. A high seed coefficient of variation indicates that the phenotypic trait varies greatly among varieties, contributes significantly to germplasm diversity, and allows for a high degree of variety differentiation (Theimer 2003; Hou et al. 2021). Regarding phenotypic variation, many studies have been conducted by previous researchers. One study found that there is abundant phenotypic variation in *Tectona grandis* seeds among provenances and families, indicating that the results of this research can effectively distinguish between different *T. grandis* varieties (Li, Huang, et al. 2024). Another study found that under different growth environments, the phenotypic variation in seed size of *Agriophyllum squarrosum* also differs, suggesting that we can use phenotypic variation in sand rice seeds to determine the surrounding environment, or, conversely, use the environment to infer the phenotypic characteristics of sand rice seeds (Zhao et al. 2022). The plasticity index of a seed is a criterion for evaluating its ability to respond to environmental stresses, and a higher index implies that the plant possesses a superior ability to self‐adjust and adapt to environmental changes (Cartelier et al. 2021). In this study, the YG‐4 seeds exhibited the lowest coefficients of variation and plasticity indices for all traits, indicating the weakest but most stable plasticity. Overall, seed length showed the greatest variation and the strongest plasticity, while seed thickness was the opposite. This is similar to the analysis of phenotypic variation coefficients of *Benincasa hispida* conducted by Qiao et al. (2014) and Guan et al. (2022). Li, Li, et al. (2024) also found similar results in the analysis of the coefficient of variation for eight phenotypic traits among 25 provenances of *Quercus mongolica* . These results can be interpreted to mean that above the gradient of environmental resource changes, *T. vernicifluum* mainly adopts the adaptive strategy of changing seed length to maintain stable seed shape. In summary, the seeds of the NLS group and ZYG group *T. vernicifluum* both performed well, with the seeds of LS‐6 exhibiting the best performance, YG‐3 having the thickest seeds, and YG‐4 demonstrating the strongest stability. Therefore, in future research or production cultivation, seeds from the NLS and ZYG groups can be prioritized as excellent germplasm resources for *T. vernicifluum* breeding and seedling cultivation. Among them, LS‐6 and YG‐3 can be given preference; if cultivation is required under different environmental conditions, the most stable YG‐4 can be considered as the first choice.

Correlation analysis responds to the degree of correlation closeness between two or more variable factors and can be used to explore the pattern of change among variables, and understanding the variation and correlation among plant traits will help to understand the adaptive strategies of plants to the environment and the mechanism of community aggregation (Ma, Wang, et al. 2024). For example, Petit et al. (2020) found that 123 *Cannabis sativa* materials showed significant variability in 28 traits related to fiber quality, varying by environment. Principal component analysis (PCA) is a multivariate statistical method, which can effectively improve the quality of breeding work and the effectiveness of parental pairing in multiobjective breeding (Pallvi et al. 2025). In this study, the phenotypic traits of *T. vernicifluum* were closely related. Seed length was extremely significantly positively correlated with seed thickness, seed breadth was extremely significantly negatively correlated with length‐to‐width ratio, and seed thickness was significantly positively correlated with length‐to‐width ratio. Among these traits, seed thickness, length‐to‐width ratio, diameter of chest, and clear length are the main components that can represent most of the information. This is similar to the findings of Huang et al. (2024) on 59 germplasm seeds of Alfalfa, in which seed phenotypic traits were mostly significantly correlated between two and two, and also to the findings of Subodh et al. (2024), in which both morphological characteristics of forest trees and seed traits showed significant correlations. In the field of research on seed phenotypic diversity, since many traits tend to follow the law of chain inheritance, it is particularly crucial to analyze the correlations between these traits, especially in the study of *T. vernicifluum* seeds, where significant correlations between different phenotypic traits are commonly found, reflecting the close links between various traits during seed growth and development. This understanding of correlation is of great value in guiding us to comprehensively assess the phenotypic traits of *T. vernicifluum* seeds using multiple indicators. In summary, most of the phenotypic traits among different provenances of *T. vernicifluum* exhibit correlations, with these traits being closely associated. Seed thickness, seed length‐to‐width ratio, diameter of chest, and clear length are the main components, which can be used as reference indicators in future selection and breeding work.

### 4.2 Screening of SSR Primers and Analysis of Genetic Diversity in *Toxicodendron vernicifluum*

Genetic diversity is a key component of biodiversity and serves as the foundation for species survival, adaptation, and evolution. A thorough understanding of the genetic diversity of a species is crucial for its conservation and utilization (Ma, Han, et al. 2024). In this study, we used 24 pairs of SSR primers to analyze the genetic diversity of 36 *T. vernicifluum* germplasm resources based on SSR‐PCR and revealed the genetic diversity level of *T. vernicifluum* germplasm resources. Among them, SSR molecular marker technology is abundant, stable, and polymorphic, which can evaluate the genetic diversity of germplasm resources in all aspects (Kumar et al. 2024). In this study, among the 36 *T. vernicifluum* family lines, the average Na was 1.826 and the average Ne was 1.517. The difference between Na and Ne is the same as that found in the study of *Citrus maxima* by Duan et al. (2025), with no significant difference. The number of alleles at the SSR loci did not differ significantly from the number of effective alleles, which indicates that the alleles were evenly distributed in the population (Kimura and Ohta 1969). This also reflects the rich genetic diversity of Yunnan *T. vernicifluum* germplasm and the relatively healthy and stable population genetic structure. PIC is often used to assess the discriminatory ability of primers and the reliability of the information they provide (Aydin et al. 2011). When PIC > 0.500, the polymorphism of the primers is high and provides rich information, which reflects the genetic diversity well; when 0.250 < PIC ≤ 0.500, the polymorphism of the primers and the amount of information they provide is high, which provides reasonable information; when PIC ≤ 0.250, the primers' polymorphism is low and provides less information (Li et al. 2020). In this study, 24 pairs of core primers with high polymorphism and good stability were screened from 160 published SSR primer pairs. The average PIC value was 0.257, which is higher than 0.250 and falls within the same range as the PIC value (0.32) of *Carthamus tinctorius* (Gaddam et al. 2025). This indicates that the primers exhibit high polymorphism and provide a considerable amount of information, making them suitable for genetic analysis or association studies within the population. Among them, the PIC values of primer M156 and primer Tox003 were as high as 0.829 and 0.784, respectively, indicating that these two pairs of primers could reflect the genetic diversity of *T. vernicifluum* well, and there were half of the primers that could provide more reasonable information, and these SSR molecular markers had higher polymorphism and more allele numbers, indicating that they have an important role in the analysis of relatedness among *T. vernicifluum* families and fingerprint identification. However, the number of amplified alleles of SSR molecular markers does not directly reflect their ability to identify *T. vernicifluum* lineages, which is in agreement with what was reported by Hameed et al. (2012). In the present study, the 24 primer pairs selected showed high polymorphism for the analyzed materials. However, the ability of these primers to maintain efficient discrimination for other materials or as the number of varieties and places of origin increases still needs to be further explored and validated. For example, the low PIC value of primer bcrs072 in this study may be due to the fact that the assessment of high and low primer polymorphism is strongly influenced by the range of *T. vernicifluum* DNA material utilized (Matsuoka et al. 2002; Lübberstedt et al. 1998). He and *I* are important indicators of species genetic diversity (Wang et al. 2015). In this study, at the species level, He and *I* were 0.230 and 0.377, respectively, and *I* was higher than the, Shannon diversity index of 0.27 (Guo et al. 2019) for *T. vernicifluum* populations in Jinnan, Shanxi, but was slightly lower than the results of the studies on genetic diversity of *T. vernicifluum* by Wang, Li, et al. (2024), Wang, Zhou, et al. (2024), and Wang et al. (2022). Apparently, the genetic resources of *T. vernicifluum* in Yunnan Province are equally rich in diversity compared with other regions. Due to its complex terrain of undulating mountains, diverse climatic conditions, and its role as a transitional zone for multiple floristic regions, Yunnan Province has formed numerous ecologically isolated natural refuges. This characteristic has enabled different populations of *T. vernicifluum* to accumulate unique genetic variations through long‐term adaptation to local environments while also avoiding homogenization caused by gene flow, thereby preserving abundant genetic resources. Which provides a solid genetic basis for further improvement of *T. vernicifluum* genetic resources.

The coefficient of population differentiation (Fst) is a genetic parameter that assesses the degree of genetic differentiation between populations; when Fst < 0.050, the degree of interpopulation differentiation is low; when 0.050 ≤ Fst < 0.150, the degree of interpopulation differentiation is moderate; when 0.150 ≤ Fst < 0.250, the degree of interpopulation differentiation is high; when Fst ≥ 0.250, the degree of genetic differentiation between populations is very high (White et al. 2007; Frankham et al. 2002). The average Fst value of the 36 *T. vernicifluum* families in this study was 0.220, which is higher than the Fst (0.01–0.15) obtained by Wang et al. (2025) in their genetic diversity analysis of *Camellia sinensis* . This indicates that there is a very high genetic differentiation within the six *T. vernicifluum* populations. In addition, similar results were obtained from the AMOVA analysis, showing that the genetic variation of *T. vernicifluum* is mainly distributed within populations. Genetic differentiation is subject to a combination of factors in a population, including resource distribution, reproductive mechanisms, gene flow, and seed dispersal (Ge 1994). Among these factors, gene flow plays a crucial role. Generally, species with high gene flow have relatively low genetic differentiation among populations. According to the value of *Nm*, gene flow was classified into three classes: 0 < Nm < 0.250 as low; 0.250 ≤ Nm < 1.000 as medium; and Nm ≥ 1.000 as high (Govindaraju 1988). When Nm ≥ 1.000, gene flow is sufficient to resist the effects of genetic drift and attenuate differentiation between populations. The Nm in this study is 1.867, which is at a high level and much greater than the Nm (0.141) of *Beta vulgaris* (Zhao et al. 2025). The high level of gene flow within *T. vernicifluum* populations results in considerable genetic differentiation within the populations. The main reason for this may be that the test population was distributed over a relatively long geographical distance, spanning both northeastern and northwestern Yunnan Province, and the geographic barrier between them impeded interpopulation gene flow. In this study, the mean value of Fis was −0.103 (Fis < 0), and the *T. vernicifluum* germplasm resources were heterozygously mated with an excess of heterozygotes. Among the six *T. vernicifluum* populations, the ZYG population has the relatively richest genetic diversity and can be prioritized as a key group for the selection of genetic materials. Combined with the phenotypic analysis results from this study, the seeds of the ZYG population as a whole performed well and are considered excellent germplasm resources for breeding and seedling cultivation. Therefore, based on comprehensive phenotypic and genetic analyses, we can draw the final conclusion that the ZYG *T. vernicifluum* population is superior. Genetic diversity is the result of a species' adaptation to its environment during the evolutionary process, and it is closely linked to factors such as the geographical distribution of the species, its life cycle, its mode of reproduction, its genetic drift, and its human activities (Abul Khayer et al. 2024). *T. vernicifluum* has a wide distribution in Yunnan Province, and the economic value of its raw lacquer and lacquer seeds contributes to the richness of its genetic diversity. *T. vernicifluum* grows at altitudes ranging from 800 to 3000 m, and in China they are widely distributed in Yunnan, Sichuan, Xizang, Shaanxi, Shanxi, and Hunan. In addition, *T. vernicifluum* are also found in countries such as India, Korea, and Japan. In order to adapt to the complex topography and diverse climatic conditions in its distribution area, *T. vernicifluum* has accumulated a large amount of genetic variation during its long evolutionary process. Because of its high economic value, humans have become the main force in seed dispersal, which has not only helped to expand the population size of *T. vernicifluum* but also enhanced the genetic diversity of *T. vernicifluum* species.

### 4.3 SSR‐Based Clustering and Genetic Structure Analysis of *Toxicodendron vernicifluum* Populations

Cluster analysis is a common tool to study the kinship and origin of germplasm resources and is a prerequisite for the conservation and utilization of germplasm resources (Zheng et al. 2023). In this study, 36 accessions of *T. vernicifluum* germplasm were divided into three groups using UPGMA clustering based on Nei's genetic distance. This result is consistent with the clustering analysis of *Mangifera indica* by Muniyappan et al. (2025), in which 30 varieties were roughly divided into three significant clusters. The clustering of individuals within *T. vernicifluum* populations was consistent; the composition of the first cluster was similar to that of the third group in the STRUCTURE analysis. The NHX and DWD populations separated first, and the remaining four populations were divided into two subgroups: ZNC and ZHZ formed one group, while ZYG and NLS formed another group. The study showed that the clustering results and geographic origin of some *T. vernicifluum* populations and lineages were not consistent, which was in agreement with the findings of Chen et al. (2021) and Li et al. (2022). It is hypothesized that the geographical origin of germplasm resources has some influence on genetic differences, but the two are not necessarily related (Wang et al. 2023). The reason for this may be that *T. vernicifluum* , as a specific plant species, has relatively stable genetic information within its population, which is key to the survival and reproduction of the species. Even if *T. vernicifluum* grows in different regions of Yunnan, as long as they belong to the same species, their basic genetic characteristics will remain the same. In addition, environmental factors such as climate and soil in Yunnan may have a selective effect on the genetic characteristics of *T. vernicifluum* . If *T. vernicifluum* from different regions grow under similar environmental conditions, they may be subject to similar environmental selection and evolutionary pressures, leading to increased similarity in genetic characteristics. For example, specific environmental factors may promote the expression of certain genes and repress the expression of others, allowing *T. vernicifluum* to develop similar genetic traits in these regions (Akhil et al. 2024). Another possible cause is genetic exchange (He et al. 2023). Although the *T. vernicifluum* of Yunnan may be distributed in more distant areas, there may be genetic exchange between them through pollination or seed dispersal by wind, animals, and other media. This exchange helps to maintain genetic diversity within populations and may also lead to some genetic similarity between *T. vernicifluum* in different regions. The results of the principal coordinate analysis (PCoA) showed both relative independence and intertwining of the six group samples and verified the reliability of the cause analysis described above.

The STRUCTURE analysis in this study indicated that the 36 *T. vernicifluum* germplasms were divided into three subpopulations, and the results were consistent with the clustering analysis. Among them, Groups 1 and 2 were distinctly differentiated from Group 3; all germplasms had mixed genetic backgrounds, involving two or three groups; the genetic composition within the same group was basically the same. This is consistent with the research results on *Kadsura coccinea* by Li, Chen, et al. (2024). It indicates that the genetic composition of *T. vernicifluum* germplasm in Yunnan Province exhibits complexity, and the *T. vernicifluum* germplasm resources collected in this region show both high genetic similarity and genetic differentiation. The phenotypic traits of different *T. vernicifluum* sources in this study vary yet also exhibit correlations, which are mutually validated by the fact that the genetic makeup of these different *T. vernicifluum* germplasms shows both similarities and differentiation. A comprehensive comparative analysis of both aspects explains the rationality of the study. In addition, there is an exchange of genetic information between these germplasm resources caused by natural or anthropogenic factors. Analyzing the causes, it may be related to the discovery of triploid *T. vernicifluum* (Shang et al. 1985) and their significant economic and medicinal value. Artificial screening of these varieties by breeders, together with the increased promotion of these varieties in the market, has led to the gradual reduction in some germplasm resources with regional characteristics. In addition, the frequent use of high‐performing parental material in production practice (Thomas et al. 2024) has led to the problem of homogenization of varieties due to the increasing homogeneity of the genetic background of the population underlying the breeding and the increased genetic similarity between varieties. Another possible reason is that the 36 *T. vernicifluum* germplasm resources collected in this study were mainly from Yunnan Province, covering most of the region in Yunnan Province, which is relatively rich in genetic diversity and representative. However, compared with the whole of China, the scope of the study is still limited, and the amount of germplasm material is small, with relatively homogeneous genetic backgrounds; therefore, most of the genetic backgrounds of *T. vernicifluum* germplasm in this study were of two or three types, which were both distinguished and related. In this study, the genetic difference between the NLS and NHX populations was the smallest, while the difference between ZNC and NHX was the largest; the difference between individuals LS‐5 and LS‐7 was small, whereas the difference between YG‐3 and HX‐2 was large. Both NLS and NHX populations were located in Nujiang Prefecture. It indicates that genotype distribution is more associated with geographical location, which is consistent with the results of Kiwuka et al. (2023) and Raskar et al. (2022) on the genetic diversity aspects of *Coffea canephora* and *Embelia ribes Burm*, and these data are closely linked to genetic similarity indexes, which further enhances the credibility of the results.

## 5 Conclusions

A total of 24 pairs of SSR primers were screened in this study, and these markers are highly informative, among which primer M156 and primer Tox003 can well reflect the genetic diversity of *T. vernicifluum* germplasm in Yunnan Province. SSR analysis revealed that the 36 *T. vernicifluum* germplasms had accumulated a large amount of genetic variation, exhibited abundant genetic diversity, and possessed a relatively complex genetic background. In addition, to improve the survival rate of *T. vernicifluum* seedlings, seeds from the NLS and ZYG populations can be prioritized, among which LS‐6 and YG‐3 are preferred choices. This research provides a certain foundation for the utilization and selection of *T. vernicifluum* parental resources. However, due to the limited number and narrow scope of *T. vernicifluum* germplasms collected in this study, as well as the relatively limited research content, the quantity and range of *T. vernicifluum* germplasm collection can be expanded in the future, and a high‐density genetic linkage map of *T. vernicifluum* family populations can be further constructed, thereby facilitating QTL mapping of phenotypic quantitative traits. At the same time, excellent characteristic gene resources can be explored through the integration of germplasm resources and genomics, providing better germplasm materials for the innovation of *T. vernicifluum* germplasm resources and the breeding of new varieties.

## Author Contributions

**Huiping Zeng:** data curation (lead), formal analysis (lead), investigation (lead), software (lead), writing – original draft (lead), writing – review and editing (lead). **Xingze Li:** data curation (lead), formal analysis (lead), investigation (lead), software (lead), writing – original draft (lead). **Jiayu Feng:** formal analysis (equal), investigation (equal), software (equal). **Cai Wang:** data curation (equal), investigation (equal), supervision (equal). **Dan Zong:** data curation (equal), investigation (equal), supervision (equal). **Tao Jiang:** data curation (equal), investigation (equal), supervision (equal). **Xinglan Wei:** data curation (equal), investigation (equal), supervision (equal). **Qiong Dong:** funding acquisition (lead), project administration (lead), resources (lead), supervision (lead), writing – review and editing (lead).

## Conflicts of Interest

The authors declare no conflicts of interest.

## Supporting information


Appendix S1.

Appendix S2.

Appendix S3.

## Data Availability

Data can be accessed via a public link for peer review at: https://osf.io/jwtaq/.
