# Supplementary material for: Phenotypic Traits, SSR Core Primer Screening, and Genetic Diversity Analysis of Toxicodendron vernicifluum From Different Seed Sources in Yunnan, China
Source: Ecol Evol. 2025 Jul 14;15(7):e71794. doi: 10.1002/ece3.71794 (PMC12259300; doi:10.1002/ece3.71794)
Supplement: Supplementary file 2 — Appendix S2. [file ECE3-15-e71794-s003.docx]

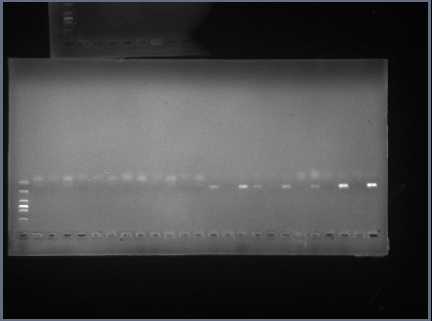


bcrs22 tox20


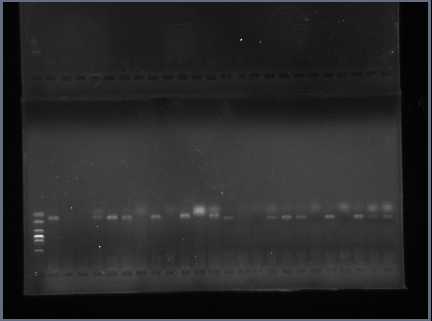


bcrs25 tox45


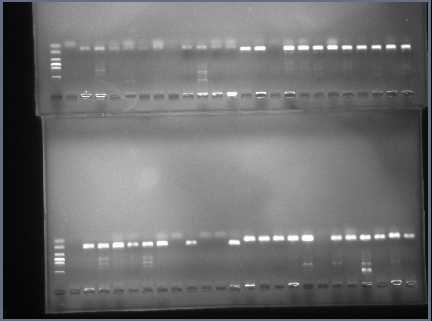


tox13 tox06

bcrs84 bcrs87


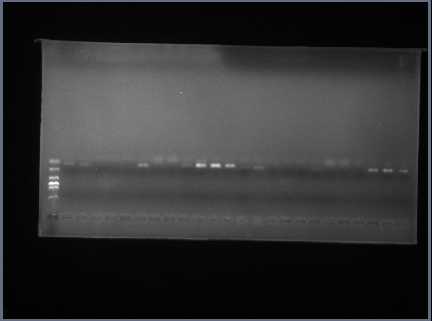


tox21 tox22


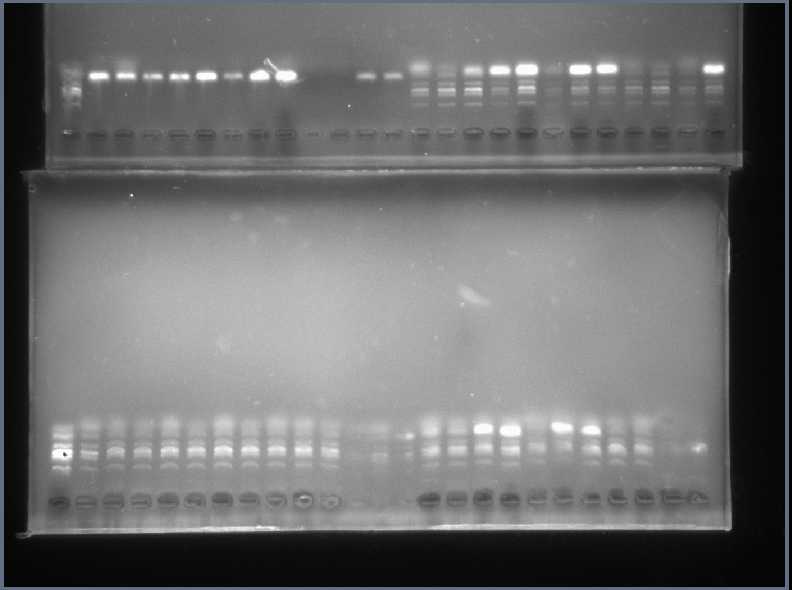


Toxo03 toxo37

Toxo14 bcrs086


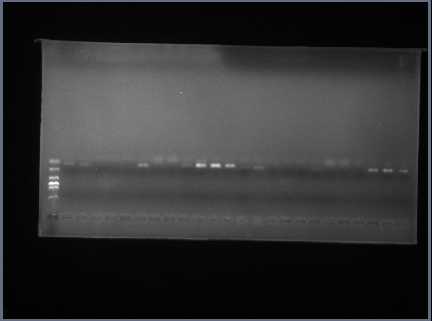


Toxo07 Toxo27


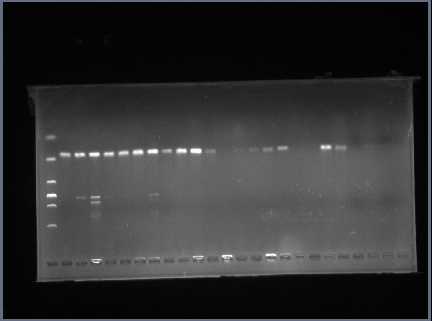


toxo46 toxo50


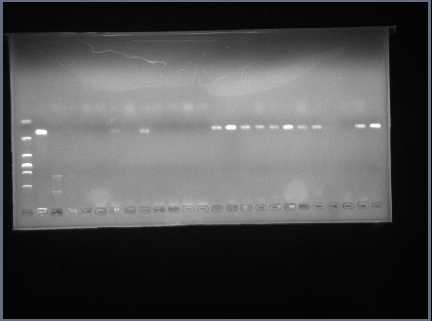


bcrs127 bcrs072


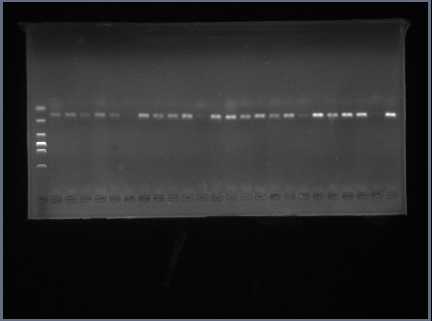


bcrs038 bcrs043


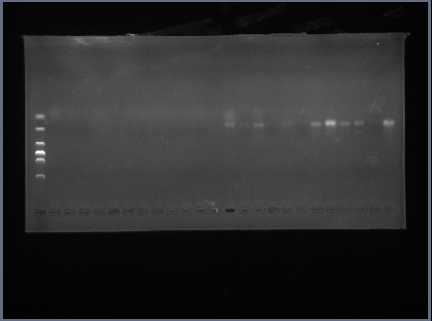


bcrs077 bcrs079


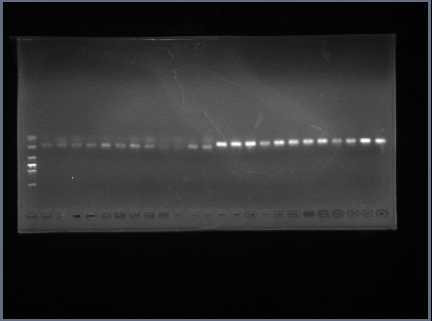


bcrs004 bcrs035


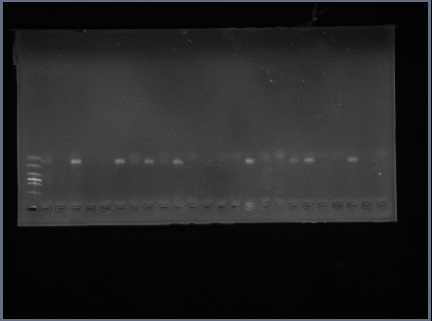


bcrs006 bcrs013


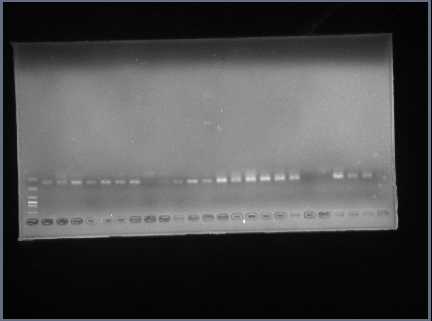


bcrs041 bcrs066


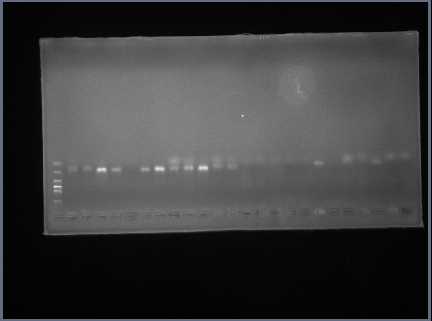


bcrs060 bcrs095


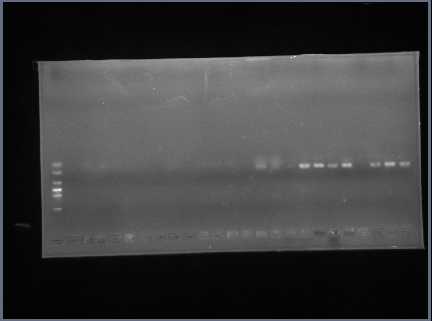


bcrs073 toxo38


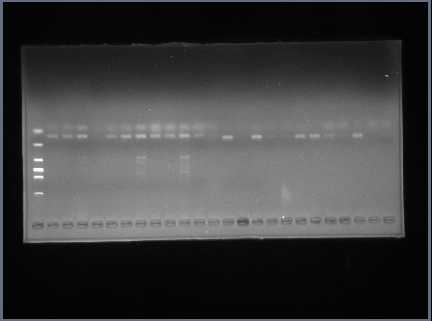


toxo28 toxo47


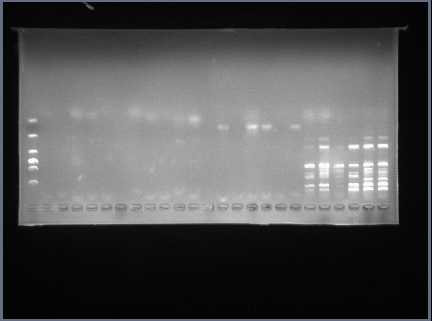


M22 M27


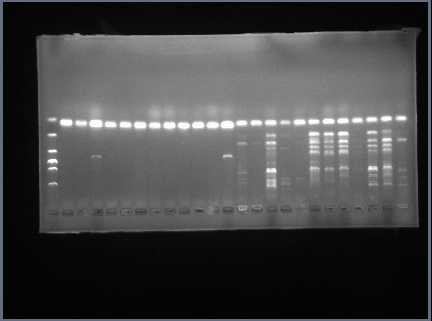


M30 M56


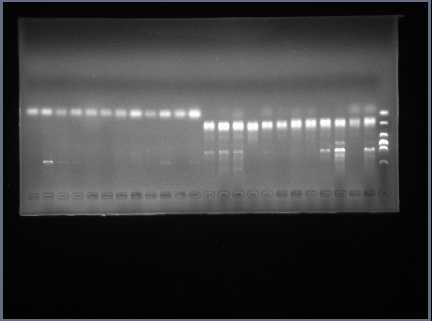


M8 M18


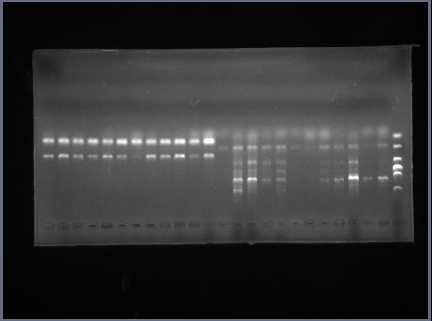


M19 M24


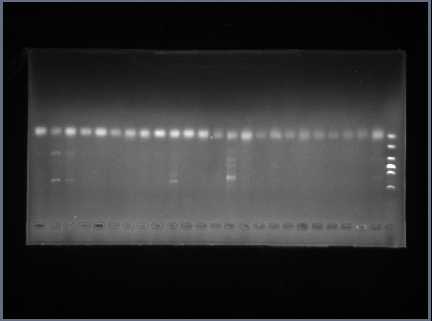


M31 M54


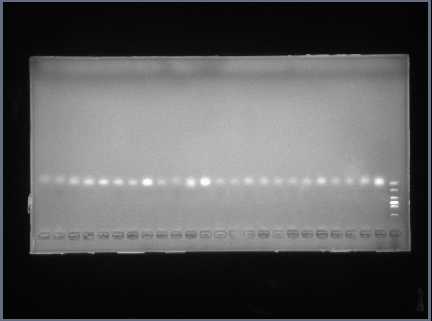


AC11 AC19


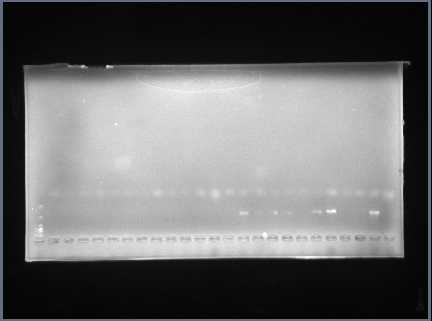


AC139 AG28


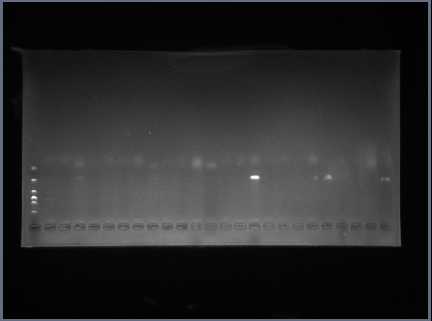


bcrs001 12co8o580


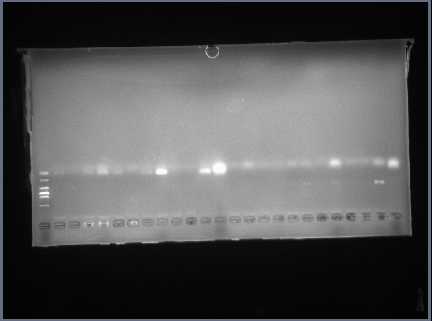


M452 AC6


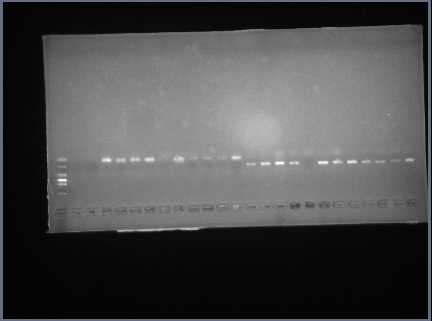


AG153 YFMS7


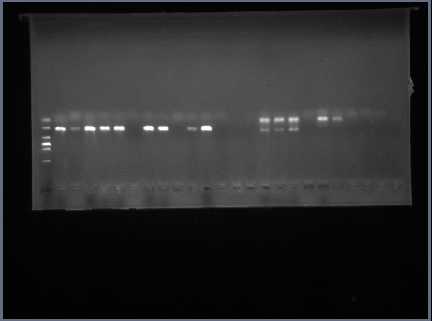


c20035 12co8o600


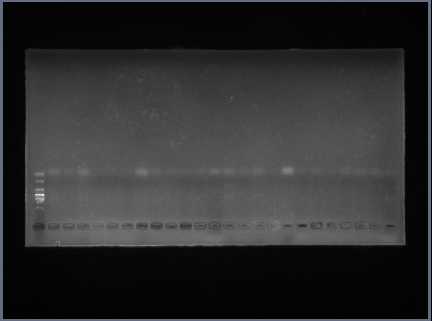


c22708 c17310


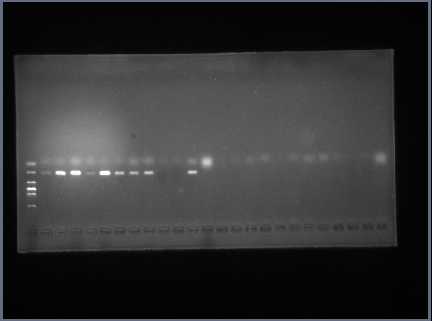


cupub21024958 cupub21319856


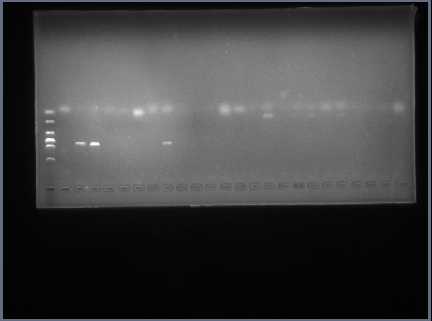


cupub21618958 cupub30618060


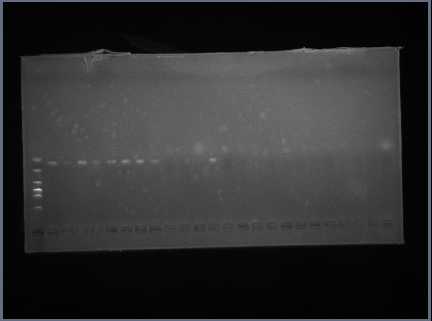


cupub30217156 cupud30727560


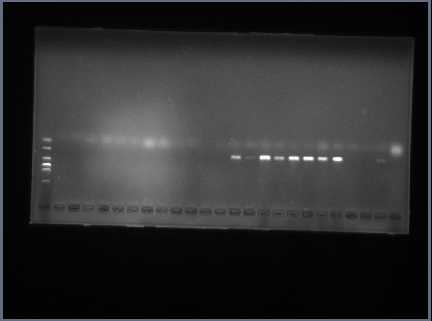


cupud00223956 cupub10831160


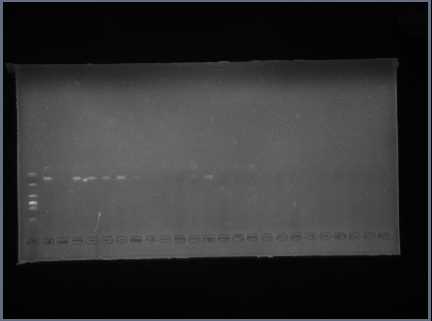


cupud11716058 cupub21718558


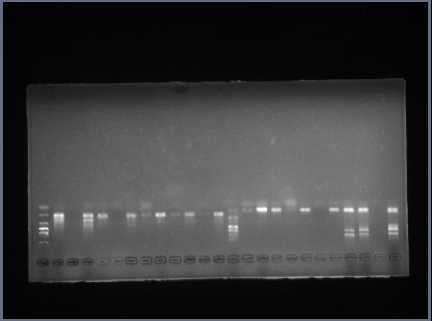


M493 c26126


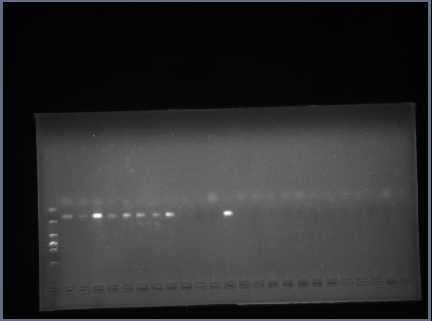


ptms7189225 ptms4215016225


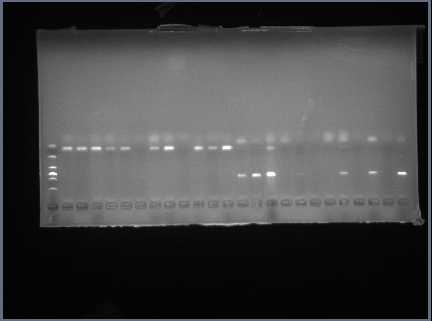


ptms313214555 YFMS20


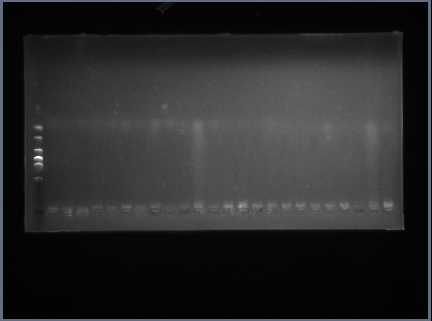


YFMS15 YFMS46


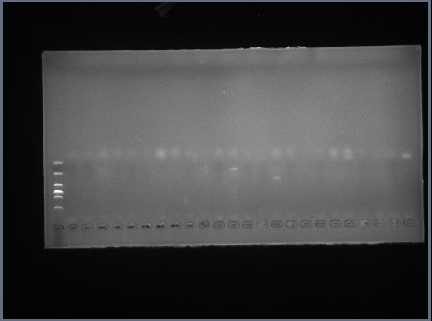


YFMS29 YFMS53


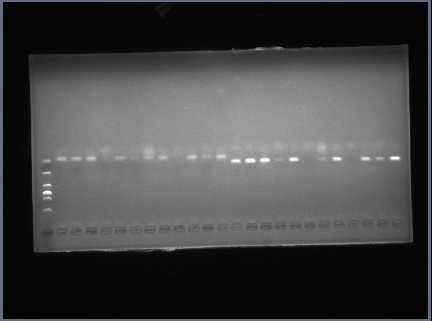


YFMS54 YFMS77


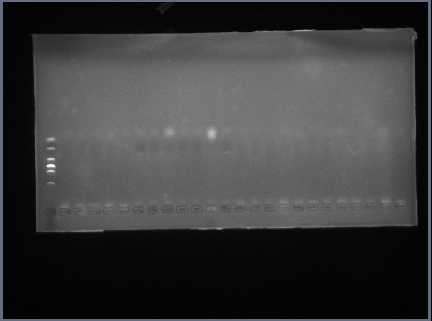


YFMS57 YFMS90


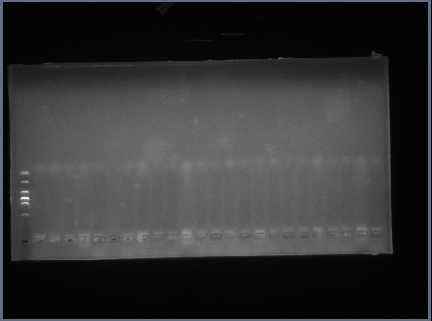


YFMS91 YFMS11


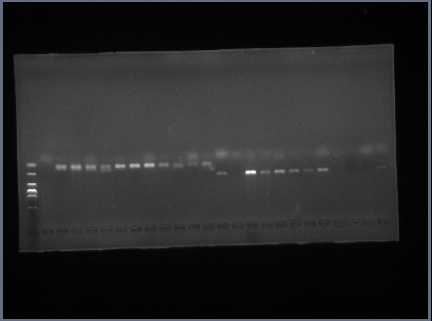


c232 ZP40


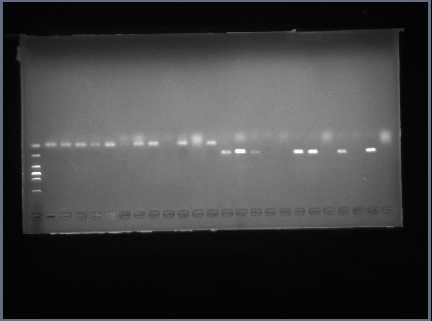


c23578 c22876


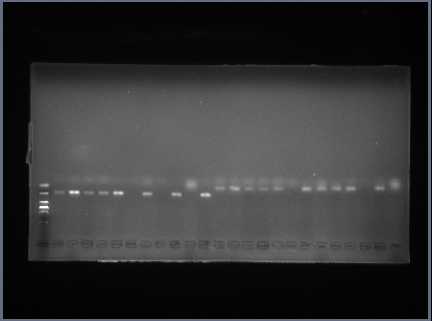


c23770 c26949


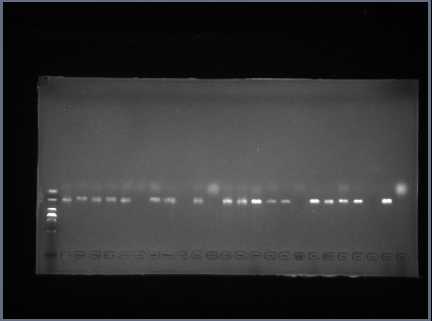


c26770 c22604


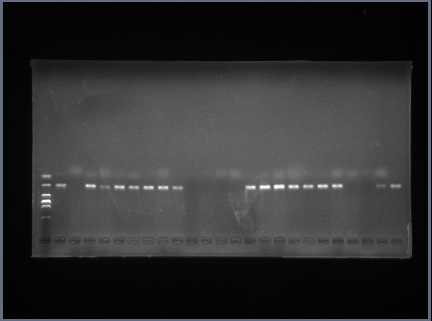


c26938 c25711


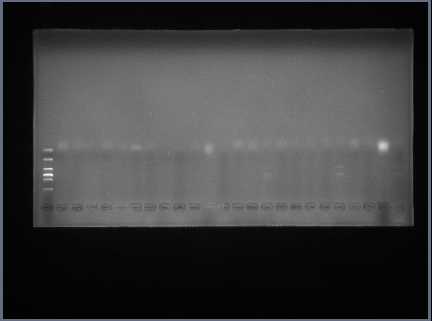


ZP1 ZP5


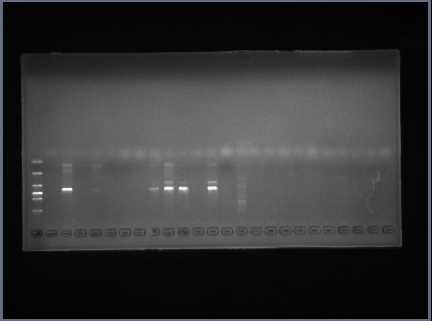


ZP7 ZP17


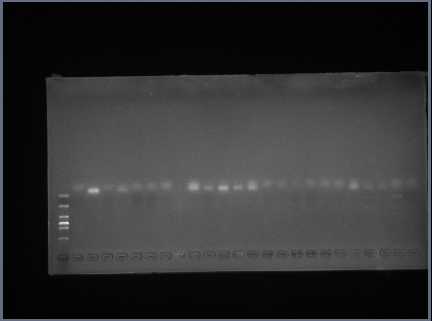


ZP8 ZP28


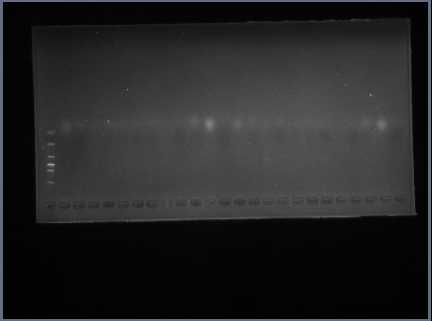


ZP13 ZP4


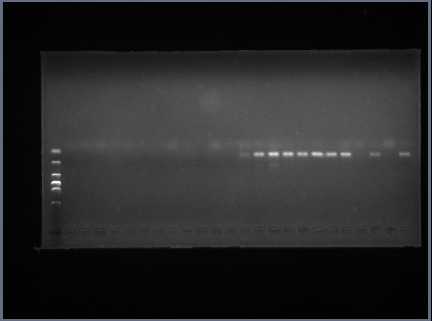


ZP22 c18564


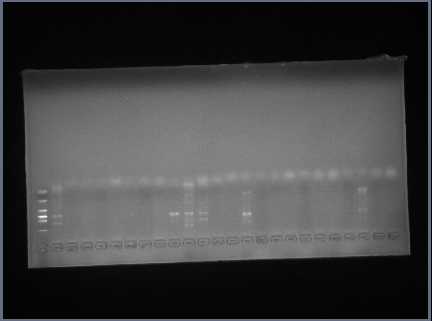


ZP32 ZP35


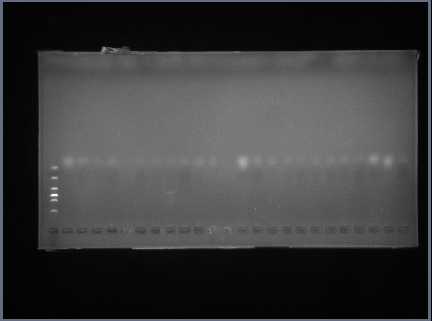


ZP41 ZP43


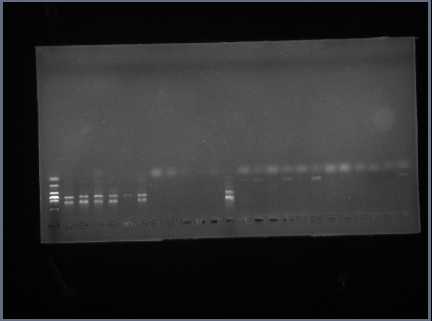


L7 ZP10


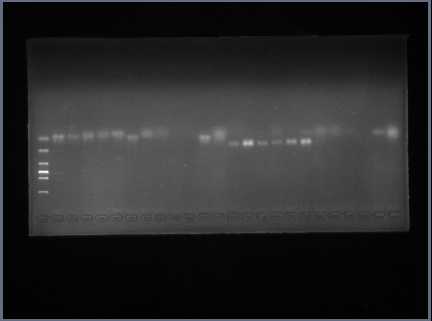


M60 M120


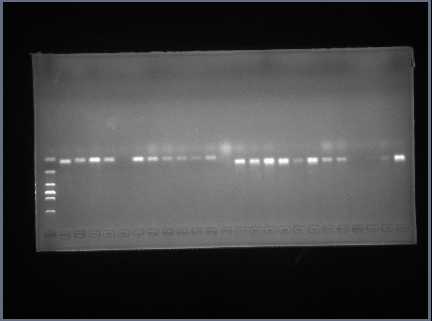


M64 M97


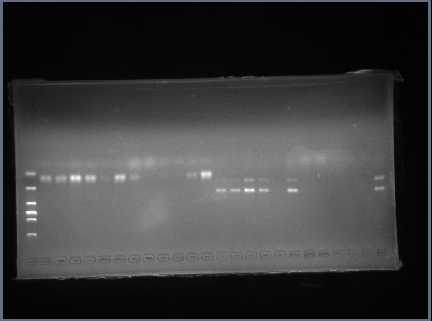


M67 ZP23


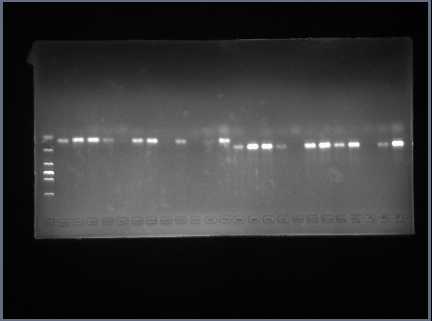


M83 M61


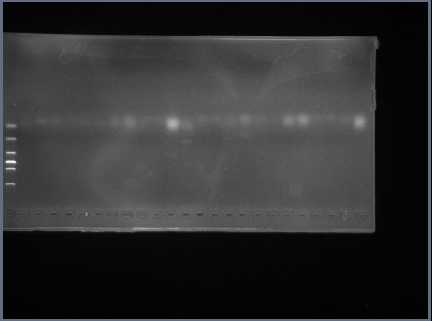


M85 M137


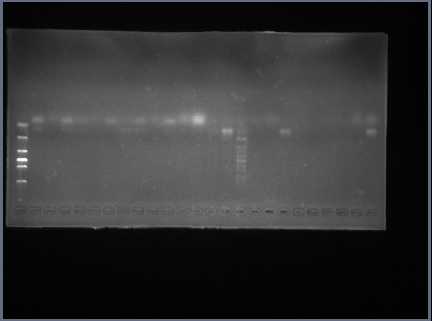


M99 M68


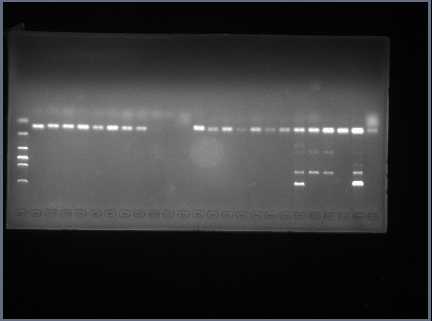


M104 M822


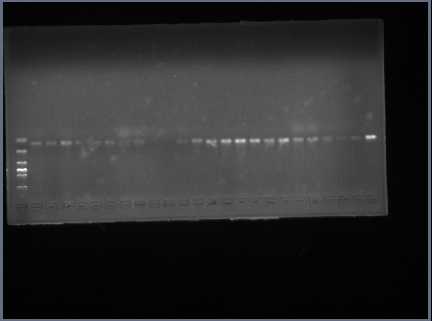


M121 M821


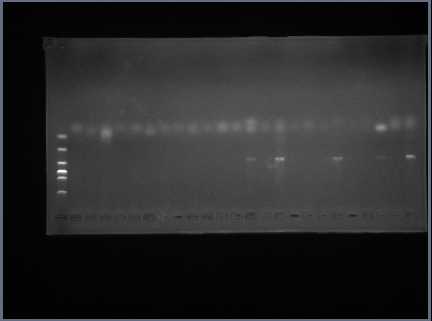


M123 M154


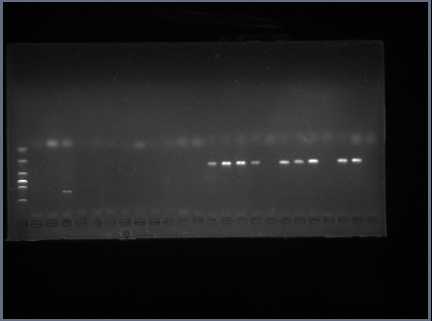


M148 M124


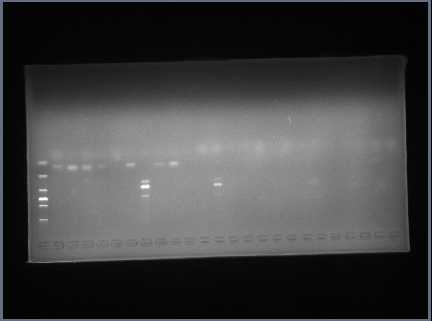


M155 ZP14


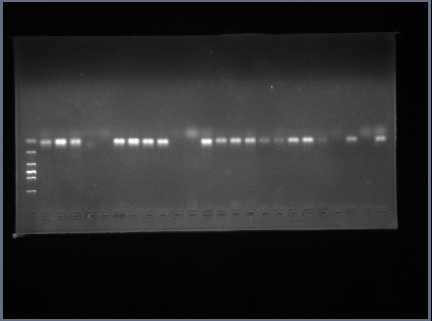


M156 M66


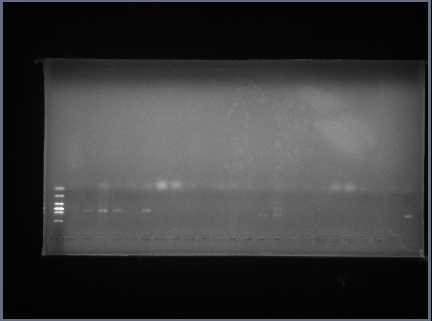


ZP24 ZP21


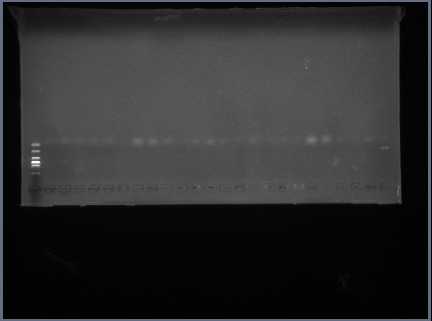


ZP25 ZP30


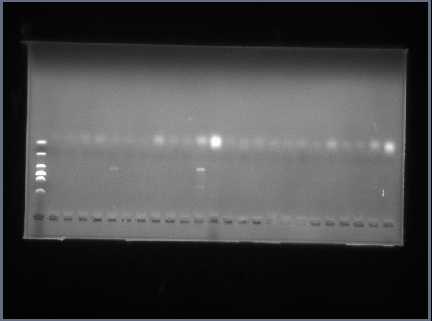


12co8o576 12co8o592


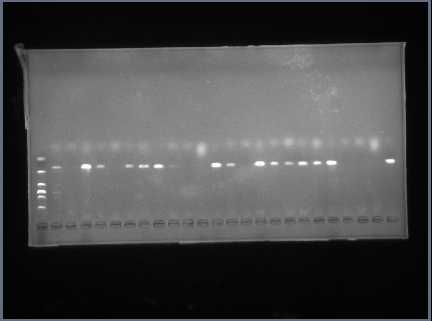


12co8o586 12co8o588


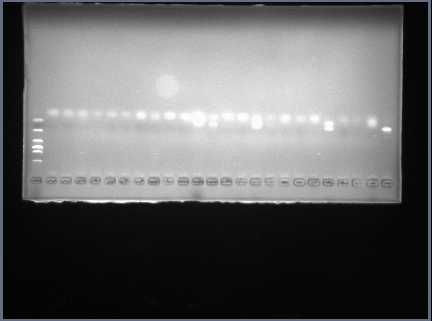


12co8o590 12co8o598


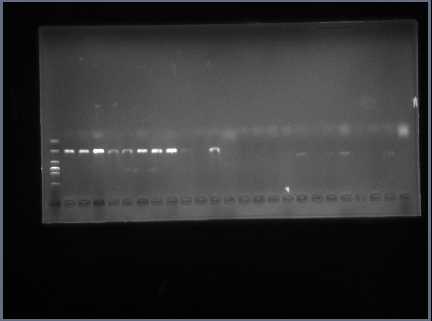


12co8o596 12co8o602


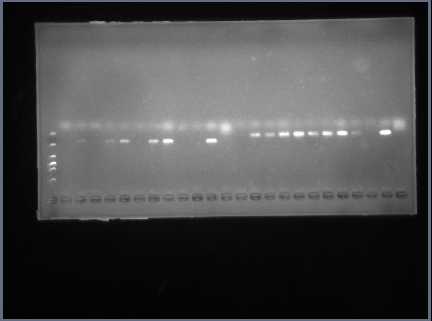


12co8o604 12co8o606


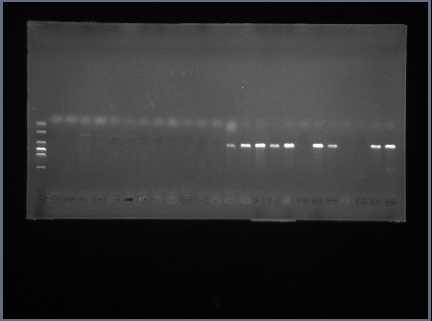


12co8o594 12co8o578


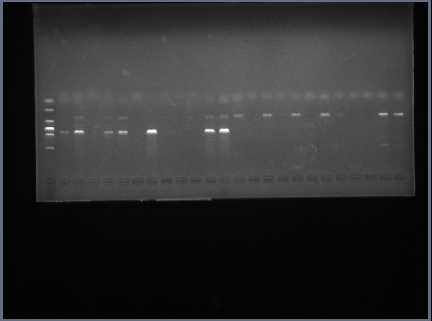


12co8o608 cupua31330558
